# Supply forecasting and profiling of urban supermarket chains based on tensor quantization exponential regression for social governance

**DOI:** 10.7717/peerj-cs.1138

**Published:** 2022-11-07

**Authors:** Dazhou Li, Bo Zhou, Chuan Lin, Jian Gao, Wei Gao, Aimin Gao

**Affiliations:** 1 College of Computer Science and Technology, Shenyang University of Chemical Technology, Shenyang, China; 2 Software College, Northeastern University, Shenyang, China; 3 Shenyang University of Technology, Shenyang, China; 4 Liaoning Chain Operation Association, Shenyang, China

**Keywords:** Supply forecasting and profiling, Urban supermarket chains, Multi-way delay embedding transform, Tensor decomposition, Exponential regression, Social governance

## Abstract

**Background:**

During the COVID-19 pandemic, the accurate forecasting and profiling of the supply of fresh commodities in urban supermarket chains may help the city government make better economic decisions, support activities of daily living, and optimize transportation to support social governance. In urban supermarket chains, the large variety of fresh commodities and the short shelf life of fresh commodities lead to the poor performance of the traditional fresh commodity supply forecasting algorithm.

**Methods:**

Unlike the classic method of forecasting a single type of fresh commodity, we proposed a third-order exponential regression algorithm incorporating the block Hankle tensor. First, a multi-way delay embedding transform was used to fuse multiple fresh commodities sales to a Hankle tensor, for aggregating the correlation and mutual information of the whole category of fresh commodities. Second, high-order orthogonal iterations were performed for tensor decomposition, which effectively extracted the high-dimensional features of multiple related fresh commodities sales time series. Finally, a tensor quantization third-order exponential regression algorithm was employed to simultaneously predict the sales of multiple correlated fresh produce items.

**Results:**

The experiment result showed that the provided tensor quantization exponential regression method reduced the normalized root mean square error by 24% and the symmetric mean absolute percentage error by 22%, compared with the state-of-the-art approaches.

## Introduction

The global epidemic of COVID-19 has had a serious impact on urban livelihoods. Urban supermarket chains are essential for the supply of urban goods in a social governance environment when policies are implemented to prevent and control the epidemic (Ihle, Bar-Nahum & Jongeneel, 2020; Viljoen & Joubert, 2019). The supply forecasting and profiling of urban supermarket chains play an indispensable role in social governance.

Fresh produce is a key issue in the forecasting and profiling of urban supermarket chains (Gibbon, 2003; Dolan & Humphrey, 2000). How to effectively break through the bottleneck of fresh goods circulation and optimise supply forecasting and profiling is of great significance for the intelligent operation of urban supermarket chains (Lin & Wu, 2011; McLaughlin, 2004). The value of fresh goods is closely linked to freshness, which is not only difficult to keep for a long time but also has high transport losses. In addition, fresh goods have various types and preservation methods, making it difficult for the supply forecasting and profiling of urban supermarket chains to be carried out efficiently and scientifically (Ilbery et al., 2004; Boerkamps, Van Binsbergen & Bovy, 2000). A large number of urban supermarket chains are still making very primary and subjective decisions on fresh produce supply forecasting and profiling that can not meet the special needs of urban supermarket chains for fresh produce (Thompson, Newsome & Commander, 2013; Bourlakis & Weightman, 2008).

Nowadays, the management of urban supermarket chains has changed from simply maximising the use of existing human and material resources to supply forecasting and profiling (Ge, Proudlove & Spring, 2004; Aburto & Weber, 2007). Supply forecasting and profiling are very important aspects of urban supermarket chains (Thompson, Newsome & Commander, 2013; Bourlakis & Weightman, 2008). Accurate supply forecasting and profiling of fresh produce can help urban supermarket chains understand consumer demand and develop more reasonable pricing and promotion plans for fresh produce (Jiang, Yao & Li, 2021; Chen, Wang & Fu, 2022).

There are two difficulties in supply forecasting and profiling fresh produce in urban supermarket chains. To address these two difficulties, we propose a tensor quantization exponential regression algorithm. Firstly, we use tensor decomposition (Ding, Qi & Wei, 2015; Mgale, Yan & Timothy, 2021) to fuse the fresh produce sales volume of multiple urban supermarket chains. The tensor decomposition technique ensures the correlation between multiple time series in a high-dimensional space (Cichocki et al., 2016; Cichocki et al., 2017; Cichocki, 2018). Secondly, the tensorized cubic exponential regression algorithm is proposed, which adapts to the cyclical nature of the fresh produce sales of urban supermarket chains. Finally, we achieve parallel computation of forecasting time series of fresh produce sales for multiple urban supermarket chains.

Traditional supply forecasting and profiling of urban supermarket chains typically use classical one-dimensional time series forecasting methods (Shukla & Jharkharia, 2011; Taylor & Fearne, 2009). Classical one-dimensional time series forecasting is used to predict trends in data by capturing patterns between historical time series data. Common time series data include commodity sales, stock prices and rainfall (Zhang & Wang, 2021). Classical one-dimensional time series forecasting methods predict future data trends by capturing the linear characteristics of historical data. However, they are not as effective for supply forecasting and profiling of urban supermarket chains (Holt, 2004; Wulff, 2017).

There are two main improvements in this research area. One is the use of combined algorithms. Jiang, Yao & Li (2021) combined the multiple linear regression algorithm with the ARIMA algorithm. Song et al. (2021) combined the SARIMA algorithm with the LSTM algorithm. Sharma et al. (2020) improved the ARIMA algorithm, and in Chai et al. (2021) improved the grey algorithm and successfully used it for supply forecasting and profiling of fresh agricultural products.

In addition, other recent research results have been noticed by researchers. Fattah et al. (2018) constructed an improved ARIMA algorithm. Sarwar et al. (2021) used LSTM neural networks for supply forecasting and profiling of urban supermarket chains. Ulrich et al. (2022) proposed a model selection algorithm for retail supply model selection, which was used in model selection for supply forecasting and profiling for the diversity of retail.

Since urban supermarket chains have a wide range of fresh goods and a short shelf life. The existing algorithms do not meet the requirements for parallel forecasting and had a higher computational cost for predicting the sales of fresh produce. We investigate the use of tensor quantization exponential regression to solve the problem of supply forecasting and profiling of fresh produce in urban supermarket chains.

## Materials & Methods

The dataset used in this article was based on Walmart commodities sales. There are 30,491 time series of length 1,941 days in this dataset. The time series were aggregated into three fresh commodities sales by region. The regions were divided into three states, California, Texas and Wisconsin. The size of the experimental dataset was 3 rows and 1,941 columns. The sample of the dataset was shown in Table S1. Each row represented a time series of one fresh commodity sale. The dataset consisted of three-time series of fresh commodity sales. Each time series contained 1,941 data, which represented the total amount of fresh commodities sold in that region on that day. The statistics covered the period from January 29 2011 to May 22 2016, which is a total of 1,941 days. The experiment used the first 1,923 days of fresh commodities sales as input data and the last 28 days as test data.

**Figure 1:**
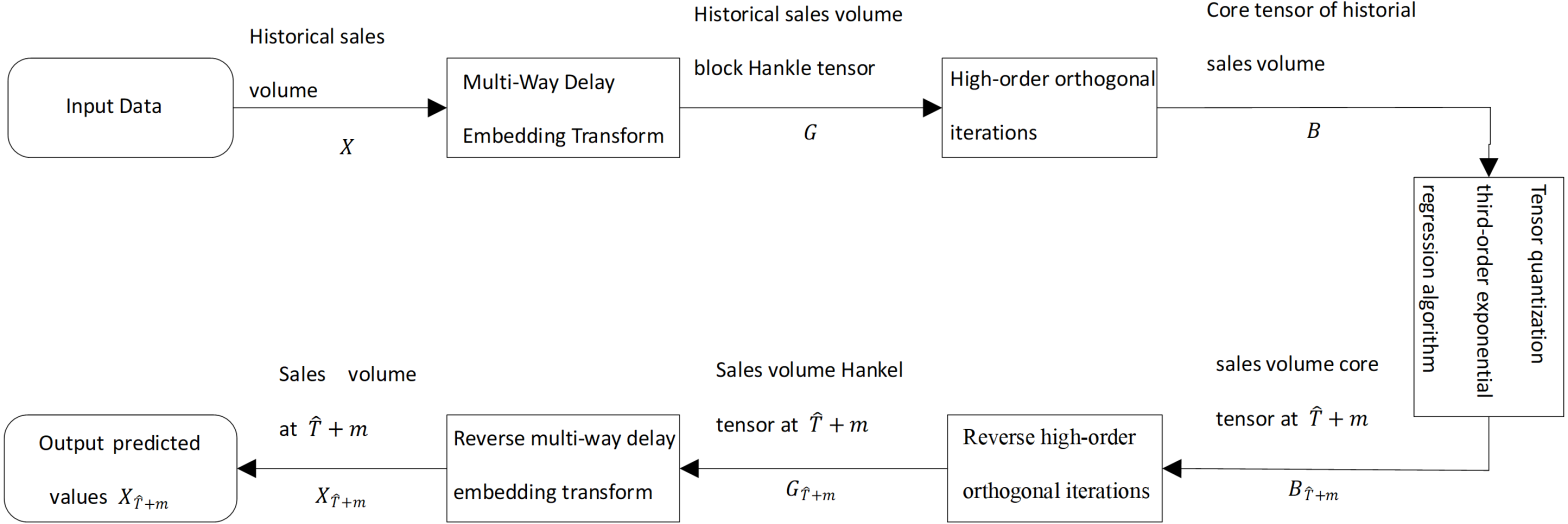
Diagram of the third-order exponential regression algorithm with block Hankle tensor.

To solve the problem of supply forecasting and profiling of fresh goods in urban supermarket chains, we proposed the tensor quantization exponential regression algorithm. As shown in Fig. 1, historical sales of fresh products in urban supermarket chains are used as input data. The input data is processed by multi-way delay embedding and transformed into a high-order Hankle tensor block (Jing et al., 2018). Then, the order orthogonal iterations are projected into the low-rank space and produce a tensor, called the core tensor. By using the cubic exponential regression algorithm for the core tensor, the future core tensor is predicted for fresh goods sales. The future core tensor is transformed into a Hankle tensor block by inverse high-order orthogonal iterations for future fresh goods sales.

We train the cubic exponential regression algorithm using the core tensor of the historical fresh produce sales, in which we exploit the correlation of multiple time series to improve the prediction accuracy. The proposed method can be adapted to the supply forecasting and profiling of fresh commodities in urban supermarket chains to provide guidance for sales planning.

We take the historical sales of fresh produce in urban supermarket chains as input data. In Fig. 1, *X* is the historical sales of merchandise in urban supermarket chains and *X* is a second-order tensor. A time series of historical sales of fresh produce is a sequence of sales of fresh produce in chronological order.

In Table S1, *X* is a second-order tensor consisting of multiple historical sales time series of fresh produce. Table S1 shows part of the data content and structure of the tensor, with each row being a historical fresh produce sales. The predicted region is represented by stated_id column, in which day_n (*n* = 1,2, …,1,941) represents the fresh produce sales on the *n*-th day in kilograms. We obtained three historical fresh produce sales time series by aggregating *X* by region. In this article, the main variables are defined as shown in Table 1.

### Multi-way delay embedding transform

In this study, we consider low-rankness in the tensor embedding space. To that point, we extend the delay embedding of time series to a multi-way delay embedding transform of the tensor. it takes the given tensor as input and outputs a high-rank Hankel tensor. Then, it recovers the higher-order tensor by a Tucker-based decomposition of the low-rank tensor. Finally, the estimated tensor is obtained by using the inverse multiplexed delayed embedding transform of the recovered higher-order tensor.

We transform the data only along the temporal dimension. The reason is the commodity adjacencies in the non-time dimension are not strongly correlated. It is also possible to arbitrarily permute the ordering between different time series. Therefore implementing the multi-way delay embedding transform in all data directions increases the computational effort, considering that order becomes larger in dimensionality. The proposed algorithm is able to support the simultaneous implementation of multi-way delay embedding transform in multiple directions, resulting in higher-order tensors.

**Table 1:** Variable definitions.

Symbol definition	Description
*X*	Historical sales volume of urban supermarket chains commodities
*S*	Duplication Matrix
*B*	Core Tensor
⊗	Kronecker product
*G*	Urban supermarket chains commodities volume block Hankle tensor
*τ*	Duplicate matrix size
${M}^{ \left( i \right) }$	*i* times exponential regression matrix
†	Moore–Penrose pseudo-inverse
*K*	Number of iterations of high-order orthogonal iterations

In Fig. S1, *X* represents the historical sales of fresh produce, and the size of *X* is *I* × *T*. The symbols *I* and *T* represent the type of fresh produce in *X* and the date of sales, respectively. The symbol *X*_*i*_ is the historical sales of all fresh produce in *X* at the date *i*-th, which is the column vector of *X*, with values in the range [1,*T*]. The symbol *G*_*i*_, *i* =1,2,…,*T*- *τ*+1, is a transformation from *X*_*i*_, *i* =1,2,…,*T*. The symbol *G* is the Hankle tensor block of historical sales of raw goods, which is a tensor with a special structure. As shown in Fig. S1, we transform *X* into *G* by a multi-way delay embedding transform.

(1) \begin{eqnarray*}\begin{array}{@{}c@{}} \displaystyle G={H}_{\tau } \left( X \right) \\ \displaystyle =Fol{d}_{ \left( I,\tau \right) } \left( X{\times }_{2}S \right) . \end{array}\end{eqnarray*}

In Eq. (1), *H*_*τ*_(*X*) means that the multi-way delay embedding transform is performed along with the second mode of *X*. The mode of a tensor is both the order and the dimension of the tensor. Since *X* belongs to *R*^*I*^^×^^*T*^, the first and second modes of *X* are *I* and *T*, which represent the category of fresh goods and the date they were sold, respectively. We only use the multi-way delay embedding transform on the second mode of *X*.

The reason is that the correlation between the sales of different types of fresh produce is usually weaker than the correlation in time. The replication matrix is denoted as *S*. A replica matrix is a matrix that is stitched together with multiple unit matrices. The process of generating a replica matrix is shown in Fig. S2. In Fig. S2, *S*^*T*^ is the transpose matrix of *S*. The symbol *I*_*τ*_ denotes a diagonal matrix of size *τ* ×*τ*. The symbol *I*_*τ*_ denotes a diagonal matrix of size *τ* ×*τ*. *S*^*T*^ is a matrix consisting of *T*- *τ*+ 1 ×*I*_*τ*_, which has the size *T*- *τ*+ 1 ×*T* as shown in Fig. S2.

The mode matrix expansion of a tensor is the process of rearranging the elements of a tensor and obtaining the mode expansion matrix (Kolda & Bader, 2009). Taking the Hankle tensor block *G* of fresh goods sales in Fig. S3 as an example, the mode matrix expansion process for *G* ∈R^*I*^^×^^*τ*^^×(^^*T*^^−^^*τ*^^+1)^ is shown in Fig. S3.

As shown in Fig. S3, the size of *G* is *I* × *τ* ×(*T*- *τ*+1). The mode-expansion matrix of *G* is labelled *G*^(^^*k*^^)^, *k* = 1,2,3. Figure S3 shows the matrix expansion process for the 1-module, 2-module and 3-modules of G^(1)^ ∈*I* × *τ*(*T*- *τ*+1), G^(2)^ ∈ *τ* ×*I* (*T*- *τ*+1), and G^(3)^ ∈*I τ* ×(*T*- *τ*+1). The modal product of a tensor is the multiplication of the modal expansion matrix of a tensor with another matrix. In Eq. (1), *X*^×^_2_*S* denotes the 2-modular product of *X*. Since *X* is a matrix, *X*^×^_2_*S* is equivalent to *XS*^*T*^, so *X*^×^_2_*S* results in a matrix of size *I* × *τ*(*T*- *τ*+1). The computational procedure of Eq. (1) can be split into *T*- *τ*+1 matrices *G*_*i*_ of size *I* ×*τ*, *i* ∈[1, *T*- *τ*+1]. *Fold*_(__*I*__,__*τ*__)_(*X* ^×^_2_*S*) in Eq. (1) denotes the composition of *G*_*i*_ into a vector *G*. The structure of *G* is shown in Fig. S1 as a Hankle tensor block of the historical sales of fresh goods, with size *I* × *τ* ×(*T*- *τ*+1).

### High-order orthogonal iterations

According to Eq. (1), *G*, a third-order Hankle tensor block, is obtained. Compared to the historical sales of fresh goods *X*, *G* has low rank and smoothness in the high dimensional space. *G* incorporates all categories of historical sales of fresh goods in *X*, *G* can be used to predict multiple time series simultaneously, thus reducing computational complexity. As the dimensionality of *G* increases, the amount of data increases exponentially. In order to reduce the computational effort, we use high-order orthogonal iterations to compress the data, which is able to preserve the valid information in the data. Figure S4 shows a schematic representation of the processing of high-order orthogonal iterations.

High-order orthogonal iterations are a tensor decomposition method, which is a generalisation of matrix orthogonal iterations to high-dimensional spaces. High-order orthogonal iterations can be used to solve for the best low-rank approximation matrix of a tensor. A low-rank approximation is an approximate representation of the original tensor by a low-rank tensor. We refer to the best low-rank approximation matrix of *G* as the core tensor, denoted by *B*. The symbol *B*_*i*_ represents the core tensor corresponding to *G*_*i*_, *i ϵ* [1, *T*- *τ*+1]. During high-order orthogonal iterations, we solve for the left singular vector of the mode expansion matrix to obtain the optimal low-rank approximation of the tensor. The process of modulo matrix expansion is shown in Figs. S3A–S3C. We solve the approximation problem for the optimal rank (*r*_1_, *r*_2_, *r*_3_) of *G*, which is equivalent to finding the tensor $\hat {G}\in {R}^{I\times \tau \times (T-\tau +1)}$. The symbol $\hat {G}$ satisfies the constraint in Eq. (2).

(2) \begin{eqnarray*}\hat {G}=argmi{n}_{W}{ \left\| G-W \right\| }_{\mathrm{F}}.\end{eqnarray*}

In Eq. (2), *W* is the loss matrix of *G* during the low-rank approximation and *rank* (*W*) is equal to (*r*_1_, *r*_2_, *r*_3_).The symbol ——*A*——_*F*_ denotes the *F*-parametrization of *A*, which is equal to ${ \left( {\mathop{\sum }\nolimits }_{i,j,k}^{n}{|}{a}_{ijk}{{|}}^{2} \right) }^{ \frac{1}{2} }$, and *a*_*ijk*_ denotes the element in tensor *A*. The *n*-modular product of a tensor is the product of each moduli expansion matrix of the tensor and the corresponding matrix. The symbol $\hat {G}$ can be written in *n*-modular product form, as shown in Eq. (3).

(3) \begin{eqnarray*}\hat {G}= \left( {U}^{ \left( 1 \right) },{U}^{ \left( 2 \right) },{U}^{ \left( 3 \right) } \right) .B.\end{eqnarray*}

In Eq. (3), *U*^(^^*n*^^)^ is an orthogonal matrix. The tensor *B* is the core tensor of *G*, and the size of *B* is (*U*^(1)^,*U*^(2)^,*U*^(3)^). The *n*-modular product of *B* is denoted as (*U*^(1)^, *U*^(2)^, *U*^(3)^), which is equivalent to *B* ×_1_*U*^(1)^ ×_2_*U*^(2)^ ×_3_*U*^(3)^. When Eq. (2) is optimised, the objective function of the high-order orthogonal iterations is obtained as illustrated in Eq. (4).

(4) \begin{eqnarray*}\min _{rank \left( \hat {G} \right) = \left( {r}_{1},{r}_{2},{r}_{3} \right) }{ \left\| G-\hat {G} \right\| }_{\mathrm{F}}=\min \nolimits { \left\| G-B{\times }_{1}{U}^{ \left( 1 \right) }{\times }_{2}{U}^{ \left( 2 \right) }{\times }_{3}{U}^{ \left( 3 \right) } \right\| }_{\mathrm{F}}.\end{eqnarray*}

Equation (4) describes a linear least squares problem. It is solved as a least squares solution of *B* ×_1_*U*^(1)^ ×_2_*U*^(2)^ ×_3_*U*^(3)^ =*G*. We use least squares to view the tensor decomposition as an optimisation problem, which can be resolved optimally by minimising the square of the error. Since *U*^(^^*n*^^)^ is a column orthogonal matrix, *B* can be described by the modal product of the tensor as shown in Eq. (5).

(5) \begin{eqnarray*}B=G{\times }_{1}{U}^{{ \left( 1 \right) }^{\mathrm{T}}}{\times }_{2}{U}^{{ \left( 2 \right) }^{\mathrm{T}}}{\times }_{3}{U}^{{ \left( 3 \right) }^{\mathrm{T}}}.\end{eqnarray*}

In Eq. (5), ${U}^{{ \left( n \right) }^{T}}$ is the inverse matrix of *U*^(^^*n*^^)^. Since *U*^(^^*n*^^)^ is a column orthogonal matrix, ${U}^{{ \left( n \right) }^{-1}}$ is equivalent to ${U}^{{ \left( n \right) }^{T}}$. The symbol *G* ×_1_ ${U}^{{ \left( 1 \right) }^{T}}$ ×_2_ ${U}^{{ \left( 2 \right) }^{T}}$ ×_3_ ${U}^{{ \left( 3 \right) }^{T}}$ represents the *n-* modulus product of *G*, which is the product of each modulus expansion matrix of *G* and the corresponding ${U}^{{ \left( n \right) }^{T}}$.

The singular value decomposition of matrices is very important in matrix calculations. Assuming that our matrix *A* is an *m* ×*n* matrix, then *A*^T^*A* is a square matrix. We find its eigenvalues and eigenvectors are (*A*^T^*A*) *v*_i_ = *λ*_i_ *v*_i_. The eigenvectors v of the matrix *A*^T^*A*, which is consist of n eigenvalues, can be obtained. Because *A*^T^*A* =*V* Σ^T^*U*^T^*U* Σ*V*^T^ =*V* Σ^T^ Σ*V*^T^ =*V* Σ^2^*V*^T^, eigenvectors v can be expanded a *n* ×*n* matrix *V*, in which each eigenvector is called the right singular vector of the matrix *A*. Similarly, (*AA*^T^) *u*_i_ = *λ*_i_*u*_i_, the matrix *U* can be obtained.

When *U* and *V* have been obtained, the matrix Σ is the final step of singular value decomposition. Since Σ is a matrix of singular values, it is only necessary to find each singular value *σ*. On the basis of *A* =*U* Σ*V*^T^, *AV* =*U* Σ*V*^T^*V*, *AV* =*U* Σ, *Av*_i_ = *σ*_i_ *u*_i_, *σ*_i_ =*Av*_i_/*u*_i_, in fact, the eigenvalue matrix Σ is equal to the square of the singular value matrix, which means that the eigenvalues and singular values satisfy the following relationship, *σ*_i_ =(*λ*_i_)^1/2^.

The higher-order singular value decomposition is an extension of the matrix singular value decomposition algorithm to the tensor (Miao, Qi & Wei, 2020; Zhang & Han, 2019), which is the process of decomposing the original tensor into smaller core tensors. The higher-order singular value decomposition of *G* is shown in Eq. (6).

(6) \begin{eqnarray*}{G}^{ \left( k \right) }={D}^{ \left( k \right) }{\sum \nolimits }^{ \left( k \right) }{V}^{{ \left( k \right) }^{\mathrm{T}}},k=1,2,3\end{eqnarray*}

In Eq. (6), *G* ^(^^*k*^^)^ is the *k*-modulus expansion matrix of the tensor *G*. The process of expanding the modulus matrix of a tensor is shown in Fig. S2. The symbols *D*^(^^*k*^^)^ and *V*^(^^*k*^^)^, *k* =1,2,3, are the matrices consisting of the left and right singular vectors of *G*^(^^*k*^^)^, respectively. The elements on the diagonal of the matrix Σ^(^^*k*^^)^, *k* =1,2,3, are called the singular values of *G* ^(^^*k*^^)^.

In Eq. (6), the sizes of *D*^(^^*k*^^)^, *k* =1,2,3, are *I* ×*I*, *τ* × *τ*, and (*T*- *τ*+1) ×(*T*- *τ*+1). For a given core tensor of size *r*_1_ ×*r*_2_ ×*r*_3_, where *r*_1_ ≤*I*, *r*_2_ ≤*τ*, and *r*_3_ = *T*- *τ*+1, the higher-order singular value decomposition of the tensor can be dimensionally reducible. We take the first *r*_1_, *r*_2_, and *r*_3_ the left singular vector corresponding to the largest singular value of *G*^(^^*k*^^)^ to obtain *U*^(^^*k*^^)^, *k* =1,2,3, which is called the truncated higher-order singular value decomposition. The sizes of *U*^(^^*k*^^)^ are *I* ×*r*_1_, *τ* ×*r*_2_, and (*T*- *τ*+1) ×(*T*- *τ*+1). To speed up the convergence of solving the core tensor, we use *U*^(^^*k*^^)^, *k* =1,2,3, as the initial value for the high-order orthogonal iterations. Table S2 shows the computation of the high-order orthogonal iterations with *G* as the solution object.

In Table S2, ⨂ denotes the Kronecker product. For example, the Kronecker product of a matrix *A* of size *m*_1_ ×*m*_2_ and a matrix *C* of size *n*_1_ ×*n*_2_ is shown in Eq. (7). The size of the result of *A* ⨂*C* is *m*_1_*n*_1_ ×*m*_2_*n*_2_.

(7) \begin{eqnarray*}A\otimes C= \left[ \begin{array}{@{}lll@{}} \displaystyle {a}_{11}B&\displaystyle L&\displaystyle {a}_{1{m}_{2}}B\\ \displaystyle M&\displaystyle O&\displaystyle M\\ \displaystyle {a}_{{m}_{1}1}B&\displaystyle L&\displaystyle {a}_{{m}_{1}{m}_{2}}B \end{array} \right] .\end{eqnarray*}

### Tensor quantization cubic exponential regression algorithm

We train a tensorised cubic exponential regression directly on the core tensor to predict the new core tensor, which not only reduces the computational effort as the core tensor size is smaller. In addition, it improves prediction accuracy by exploiting the interrelationships between multiple time series in the model construction process. Existing tensor models all constrain the mapping matrix in the direction of each data dimension. We only relax the constraints in the time dimension. This approach better captures the intrinsic correlation between the series. At the same time, the tensorization of the cubic exponential regression algorithm makes it possible to deal directly with multidimensional data, so the core tensor is necessary to achieve tensor computation.

The cubic exponential regression is an improvement on the primary and the quadratic exponential regression algorithms. It adds additional seasonal information over primary and quadratic exponential regression, which is more applicable to seasonal variation time series. The exponential regression adds seasonal information that is more applicable to seasonally varying time series. Seasonality is necessary for the cubic exponential regression forecasting algorithm. Non-seasonal series cannot be predicted by this method. the predictive strength of cubic exponential regression is related to the stability of the historical data. If there is a seasonal pattern in the historical data, the algorithm is able to capture the pattern well, otherwise, there will be a large error.

The cubic exponential regression consists of three components: the smoothing coefficient, the trend value and the seasonal component. They are the three important elements of seasonal information. If a time series repeats itself at a certain interval, then this interval is called a season. This time series is then seasonal. The seasonal length is the number of data points within a cycle of change in the series. Each point in each season is a component of seasonality.

The tensor quantization cubic exponential regression algorithm is able to predict future demand for fresh produce based on the core tensor of historical sales of fresh produce in urban supermarket chains. Compared with the classical cubic exponential regression algorithm, the tensor quantization cubic exponential regression algorithm is not only able to predict multiple categories of fresh produce sales simultaneously but also to find the correlation between multiple time series, thus improving the prediction accuracy. Figure S5 shows a diagram of the tensor quantization cubic exponential regression algorithm calculation.

The cubic exponential regression algorithm is to perform another exponential smoothing on the quadratic exponential smoothing, which corrects the predicted values of the quadratic exponential smoothing. The prediction results can adequately reflect the cyclicality of the demand for fresh commodities in urban supermarket chains. Our proposed tensor quantization cubic exponential regression algorithm is shown in Eq. (8).

(8) \begin{eqnarray*} \left\{ \begin{array}{@{}r@{}} \displaystyle {M}_{i}^{ \left( 1 \right) }=\alpha {B}_{i}+ \left( 1-\alpha \right) {M}_{i-1}^{ \left( 1 \right) }\\ \displaystyle {M}_{i}^{ \left( 2 \right) }=\alpha {M}_{i}^{ \left( 1 \right) }+ \left( 1-\alpha \right) {M}_{i-1}^{ \left( 2 \right) }\\ \displaystyle {M}_{i}^{ \left( 3 \right) }=\alpha {M}_{i}^{ \left( 2 \right) }+ \left( 1-\alpha \right) {M}_{i-1}^{ \left( 3 \right) } \end{array}. \right. \end{eqnarray*}

In Eq. (8), *α*, 0<*α*<1, is the regression coefficient in the tensor quantization cubic exponential regression algorithm. The symbol *B* ∈ *R*^*r*_1_×*r*_2_×(*T*−*τ*+1)^ is the input data to the tensor quantization cubic exponential regression algorithm. As shown in Fig. S5, *B* can be viewed as a time series of historical fresh produce sales consisting of *B*_*i*_. The symbol *B*_*i*_. is the core tensor of historical fresh produce sales for urban supermarket chains at the time *i*, and the size of *B*_*i*_ is *r*_1_ ×*r*_2_. The symbols ${M}_{i}^{ \left( 1 \right) }$, ${M}_{i}^{ \left( 2 \right) }$, and ${M}_{i}^{ \left( 3 \right) }$ are matrices obtained by subjecting *B*_*i*_ to primary, secondary, and tertiary exponential regression.

We use the tensor quantization cubic exponential regression algorithm to solve for the core tensor of fresh goods sales on the future *m* day, named ${B}_{\hat {T}+m}$, $\hat {T}$ = *T*- *τ*+1. Because the cubic exponential regression algorithm reflects the linear relationship between the input data and the exponential regression values, we can use the tensor quantization cubic exponential regression algorithm for *B* as shown in Eq. (8). It is a special kind of weighted average analysis method. The symbol ${B}_{\hat {T}+m}$ is calculated as in Eq. (9).

(9) \begin{eqnarray*}{B}_{\hat {T}+m}={a}_{\hat {T}}+{b}_{\hat {T}}m+ \frac{1}{2} {c}_{\hat {T}}{m}^{2}.\end{eqnarray*}

In Eq. (9), *m* is a positive integer and is equal to or greater than 1. The symbols ${a}_{\hat {T}}$, ${b}_{\hat {T}}$, and ${c}_{\hat {T}}$ can be obtained by Eq. (10).

(10) \begin{eqnarray*} \left\{ \begin{array}{@{}r@{}} \displaystyle {a}_{\hat {T}}=3{M}_{\hat {T}}^{ \left( 1 \right) }-3{M}_{\hat {T}}^{ \left( 2 \right) }+{M}_{\hat {T}}^{ \left( 3 \right) }\\ \displaystyle {b}_{\hat {T}}= \frac{\alpha }{2{ \left( 1-\alpha \right) }^{2}} \left[ \left( 6-5\alpha \right) {M}_{\hat {T}}^{ \left( 1 \right) }- \left( 10-8\alpha \right) {M}_{\hat {T}}^{ \left( 2 \right) }+ \left( 4-3\alpha \right) {M}_{\hat {T}}^{ \left( 3 \right) } \right] \\ \displaystyle {c}_{\hat {T}}= \frac{\alpha }{{ \left( 1-\alpha \right) }^{2}} \left[ {M}_{\hat {T}}^{ \left( 1 \right) }-2{M}_{\hat {T}}^{ \left( 2 \right) }+{M}_{\hat {T}}^{ \left( 3 \right) } \right] \end{array}. \right. \end{eqnarray*}

### Reverse high-order orthogonal iterations

Reverse high-order orthogonal iterations are the inverse of high-order orthogonal iterations. The symbol ${B}_{\hat {T}+m}$ need to be converted into a Hankle tensor block ${G}_{\hat {T}+m}$ for fresh merchandising by reverse high-order orthogonal iterations. Figure S6 shows a diagram of the conversion of ${B}_{\hat {T}+m}$ to ${G}_{\hat {T}+m}$.

The size of ${B}_{\hat {T}+m}$ is *r*_1_ ×*r*_2_ in Fig. S6 and is transformed into the Hankle tensor block of fresh commodities sales ${G}_{\hat {T}+m}$ at the corresponding moment by the reverse high-order orthogonal iterations. Equation (11) is the formula to get ${G}_{\hat {T}+m}$.

(11) \begin{eqnarray*}{G}_{\hat {T}+m}={B}_{\hat {T}+m}{\times }_{1}{U}^{ \left( 1 \right) }{\times }_{2}{U}^{ \left( 2 \right) }.\end{eqnarray*}

In Eq. (11), *U* ^(^^*n*^^)^, *n* =1,2, is the *U*^(^^*n*^^)^, *n* =1,2,3, from Eq. (4). Since the tensor ${B}_{\hat {T}+m}$ has only two modes, we take only the *U*^(^^*n*^^)^, *n* =1,2, on the corresponding mode for inverse high-order orthogonal iterations. *U*^(^^*n*^^)^ is defined as in Eq. (5). The magnitudes of *U*^(1)^ and *U*^(2)^ are *I* ×*r*_1_ and *τ* ×*r*_2_, respectively. According to Eq. (11), the magnitude of ${G}_{\hat {T}+m}$ is *I* ×*r*.

### Reverse multi-way delay embedding transform

According to the structure of Fig. 1, ${G}_{\hat {T}+m}$ must undergo a reverse multi-way delay embedding transform to obtain the forecast of fresh produce sales at the time $\hat {T}+m$, which contains the forecast of all types of fresh produce sales at the time $\hat {T}+m$. The reverse multi-way delay embedding transformation process is shown in Fig. S7. Equation (12) demonstrated reverse multi-way delay embedding transform.

(12) \begin{eqnarray*}\begin{array}{@{}c@{}} \displaystyle \hat {X}={H}_{\tau }^{-1} \left( \hat {G} \right) \\ \displaystyle =Unfol{d}_{ \left( I,\tau \right) } \left( \hat {G} \right) {\times }_{2}{O}^{\dagger }. \end{array}\end{eqnarray*}

In Eq. (12), the Hankle tensor block for fresh goods sales $\hat {G}$ consists of *G* and ${G}_{\hat {T}+m}$. The data structure of $\hat {G}$ is shown in Fig. S8, with a size is *I* × *τ* ×(*T*- *τ*+2). The operator † denotes the Moore–Penrose generalised inverse. The symbol *O* denotes the replication matrix and the structure of *O*^*T*^ is shown in Fig. S8. In Fig. S8, *I*_*τ*_ is a diagonal matrix of size *τ* ×*τ*, as shown in Fig. S3. *O*^*T*^ is a replica matrix consisting of *T*- *τ*+2 times *I*_*τ*_ of size (*T+* 1) × *τ*(*T*- *τ*+1). The formula for *O*^†^ is (*O*^*T*^*O*)^−1^*O*^*T*^, and the size of *O*^†^ is also (*T+* 1) × *τ*(*T*- *τ*+1).

In Eq. (12), ${H}_{\tau }^{-1}$ is the inverse function of *H*_*τ*_. The symbol *Unfold*_(__*I*__,__*τ*__)_($\hat {G}$) denotes the expansion of $\hat {G}$, which is illustrated in Fig. S9. In Fig. S9, *Unfold*_(__*I*__,__*τ*__)_($\hat {G}$) represents the Hankle tensor block $\hat {G}$ for fresh produce sales expanded into a matrix *R*, *R* ∈*I* × *τ*(*T*- *τ*+2). $\hat {X}$ is the 2-module product of *R* and *O*^†^. The fresh produce sales $\hat {X}$ are composed of historical sales *X* and sales forecasts ${X}_{\hat {T}+m}$ at the moment in time $\hat {T}+m$. The structure of $\hat {X}$ is shown in Fig. S10.

## Results

The experiments were performed on a Windows system. We used an RTX2060 graphics card, which has 6G of video memory. The programming language used was Python, version 3.7.3. The dataset we used was based on the Walmart fresh produce sales public dataset, aggregating 30491 time series of length 1,941 by region into three fresh produce sales time series. The regions are divided into California, Texas and Wisconsin. The size of our dataset is three rows and 1,941 columns. In Fig. S11, the horizontal axis represents the date, from day 1 to day 1,941. The vertical axis represents the volume of fresh commodities sold. The red line, the yellow line, and the grey line show the trend in daily sales of all fresh commodities in California, Texas, and Wisconsin. Each time series contains 1,941 data, each representing the total amount of fresh produce sold in that region on that day. The statistics cover a total of 1,941 days from January 29, 2011, to May 22, 2016. We used the first 1,923 days of fresh produce sales as input data and the last 28 days as test data.

### Evaluation metrics

The evaluation metrics of the experiment were evaluated using the Symmetric Mean Absolute Percentage Error, Normalized Root Mean Square Error and R-squared (Estrada et al., 2020; Kong et al., 2022a; Kong et al., 2022b; Memic et al., 2021). The abbreviations were SMAPE, NRMSE and R2 (Chicco, Warrens & Jurman, 2021; Zhu et al., 2022).

Equation (13) was the formula of SMAPE.

(13) \begin{eqnarray*}\text{SMAPE}= \frac{1}{n} \sum _{i=1}^{n} \frac{ \left\vert {\hat {y}}_{i}-{y}_{i} \right\vert }{ \left( \left\vert {\hat {y}}_{i} \right\vert - \left\vert {y}_{i} \right\vert \right) /2} \end{eqnarray*}

Equation (14) was the formula of NRMSE.

(14) \begin{eqnarray*}\text{NRMSE}= \frac{\sqrt{ \frac{1}{N} \sum _{i=1}^{N}{ \left\vert {y}_{i}-{\hat {y}}_{i} \right\vert }^{2}}}{{y}_{max}-{y}_{min}} \end{eqnarray*}

Equation (15) was the formula of R^2^.

(15) \begin{eqnarray*}{\mathrm{R}}^{2}=1- \frac{\sum _{i=1}^{n}{ \left( {y}_{i}-{\hat {y}}_{i} \right) }^{2}}{\sum _{i=1}^{n}{ \left( {y}_{i}-\overline{y} \right) }^{2}} .\end{eqnarray*}

In Equations (13)–(15), *n* indicates the number of days of test data. The symbol *y*_*i*_ indicates real fresh produce sales. The symbols *y*_*max*_ and *y*_*min*_ denote the maximum and minimum values, respectively. The symbol ${\hat {y}}_{i}$ denotes the predicted value of raw commodity sales for the proposed tensor quantization exponential regression algorithm. The values of SMAPE, NRMSE and R^2^ are all in the range of 0 to 1 (Zhang et al., 2016). When the values of SMAPE, NRMSE, and R^2^ are closer to 0, 0, and 1, the proposed method is more effective in prediction.

### High-order orthogonal iterations number selection

The loss function generally refers to the error between the predicted and true values of a single sample. The individual cost function generally refers to the error between a single batch or the entire training set and the true value (Zhou et al., 2017). In practice, the loss function refers to the overall situation. In the absence of overfitting, the goal is to minimise the loss function, with a smaller loss indicating that the predicted value is closer to the true value. Since the loss function is a direct calculation of the batch, the returned loss is a vector of dimensional batch size.

During the computation of the proposed tensor quantization exponential regression algorithm for the high-order orthogonal iterations method, we compared the magnitude of the *loss* values under different *K* in order to select the optimal number of iterations *K*. The F-norm in Eq. (2) is *loss*, as shown in Eq. (16).

(16) \begin{eqnarray*}\begin{array}{@{}c@{}} \displaystyle loss={ \left\| W \right\| }_{\mathrm{F}}\\ \displaystyle ={ \left\| G-\hat {G} \right\| }_{\mathrm{F}}. \end{array}\end{eqnarray*}

In Eq. (16), *W* and *G* are the loss matrix and the historical core tensor of fresh produce sales, respectively. The value of *loss* is normalised by using a deviation normalisation, as shown in Eq. (17).

(17) \begin{eqnarray*}los{s}_{i}^{\mathrm{\ast }}= \frac{los{s}_{i}-los{s}_{min}}{los{s}_{max}-los{s}_{min}} .\end{eqnarray*}

**Figure 2:**
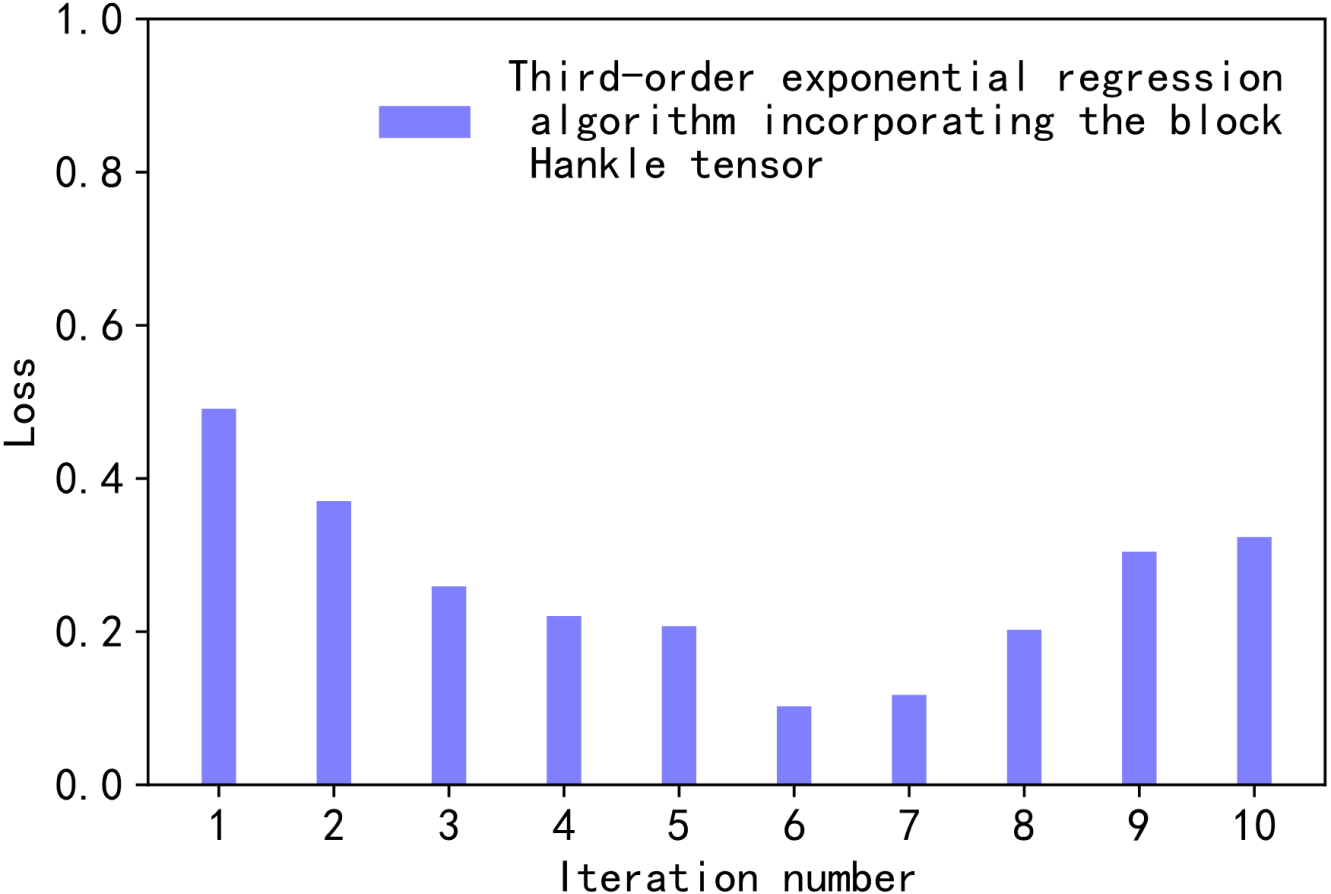
Loss values.

In Eq. (17), $los{s}_{i}^{\ast }$, *loss*_*min*_, and *loss*_*min*_ are the normalised values, the maximum value, and the minimum value of *loss*_*i*_, for a given high-order orthogonal iterations *K*. In our comparison, the values of *K* range from 1 to 10. Therefore, the value of *i* from [1,10] in Eq. (17). Figure 2 illustrates the normalised loss value *versus* the number of iterations.

We chose Eqs. (16), (17) as the loss function for the proposed method based on the subject under study. Equations (16), (17) are the more commonly used loss functions. The error curve is characterised as smooth, continuous, and derivable. Therefore, the gradient descent algorithm can be used to find the minimum value of the loss function. In addition, the gradient of the error curve decreases as the error decreases. It facilitates the convergence of the function. Even at a fixed descent gradient, the loss function can be minimised relatively quickly. Equation (16) has a special property. When the difference between *G* and $\hat {G}$ is too large, it increases error. Equation (16) imposes a larger penalty for larger errors and a smaller penalty for smaller errors. The proposed model will be more biased towards larger penalties. If there are outliers in the sample, Eq. (17) assigns higher weights to the outliers.

In Fig. 2, the horizontal axis represents the process of changing the value of the number of iterations *K*, *K* ∈ [1,10]. The vertical axis is the normalised loss value *loss*^∗^. As the value of *K* decreased from 1 to 6, the value of *loss*^∗^ decreased. When *K* was 6, *loss*^∗^ took a very small value of 0.10245697. When *K* was greater than 6, the value of *loss*^∗^ oscillated over a wide range. Since the smaller *loss*^∗^ the smaller the loss in tensor decomposition, *K* was taken to be 6 in this article.

### Regression factor selection

In Eq. (8), we need to determine the value of the smoothing coefficient *α*, 0<*α*<1, to implement the tensor quantization cubic exponential regression algorithm. The *α* value is chosen subjectively, with larger values indicating a greater weighting of more recent data in the prediction of the future. When the time series is relatively smooth, *α* is taken as a smaller value. It is possible to ignore the effect of the value of *α* on future forecasts. The *α* is generally determined by first making an approximate estimate based on experience. When the time series is relatively smooth, the chosen *α* value is between 0.05 and 0.20. When the time series is volatile, but the long-term trend does not change significantly, the chosen *α* value is between 0.10 and 0.40. When the time series is highly volatile and the long-term trend is significant upward or downward, the *α* value chosen is between 0.60 and 0.80. When the time series is a typical upward or downward series, which satisfies the additive model, the *α* value chosen is between 0.60 and 1.

**Figure 3:**
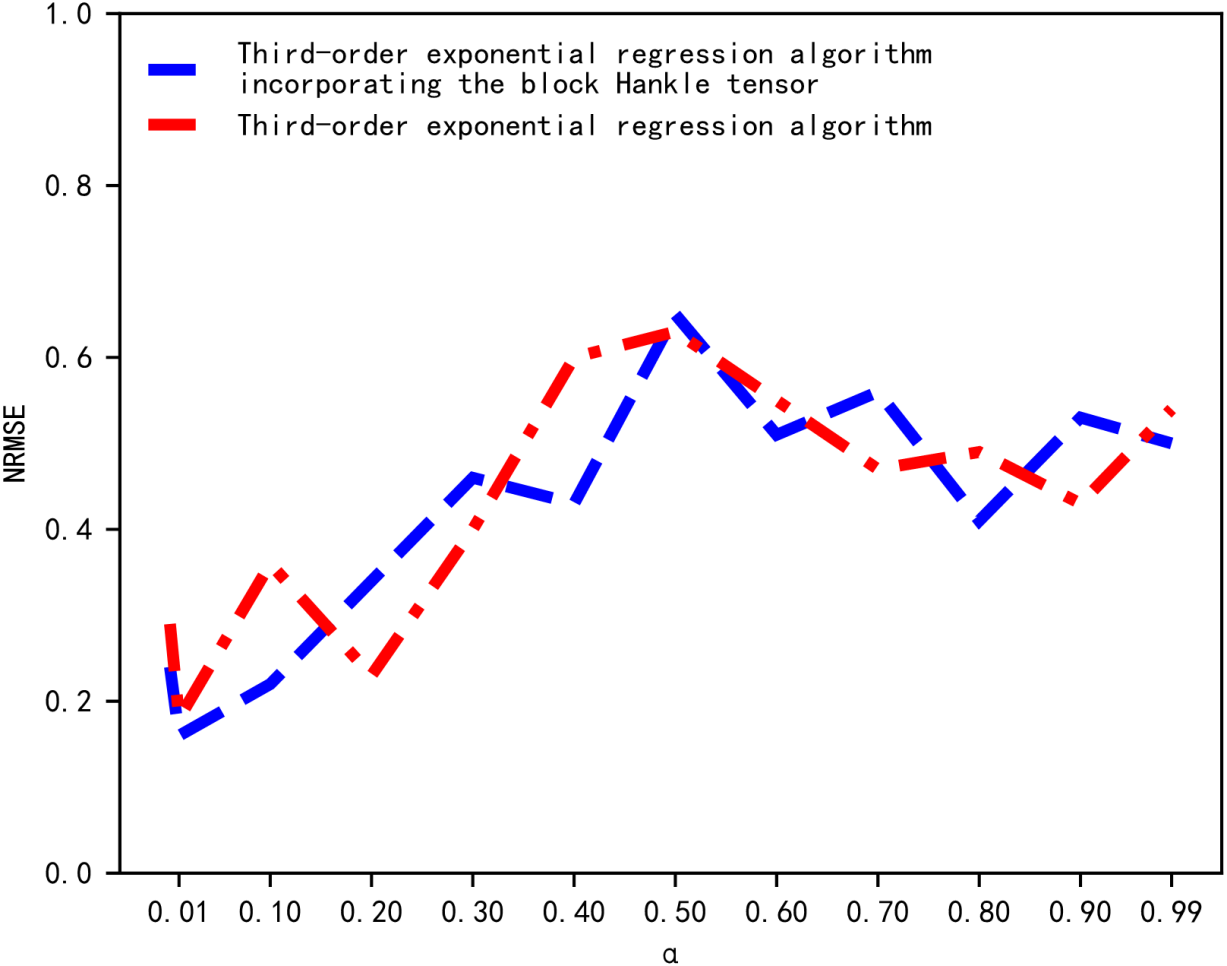
Comparison of NRMSE.

The standard error of prediction under different values of *α* is then compared through a multiple experiment process and the *α* value with the smallest error is chosen. We determined the value of *α* by comparing the magnitude of the NRMSE corresponding to different values of *α*. The results of the NRMSE comparison are shown in Fig. 3. In Fig. 3, the horizontal and vertical axes represent the value of *α* and NRMSE, respectively. When *α* was equal to 0.01, the NRMSE of the cubic exponential regression algorithm and the proposed tensor quantization exponential regression algorithm both obtained the minimum value. Therefore, the smoothing coefficient *α* was chosen to be 0.01.

### Analysis of experimental results

Tables S3 and S4 show the values of NRMSE and SMAPE for predictions on the test data, using the cubic exponential regression algorithm and the proposed tensor quantization exponential regression algorithm, respectively.

From Tables S3 and S4, it was demonstrated that the proposed tensor quantization exponential regression algorithm had the best NRMSE and SMAPE for the prediction results in California, Texas, and Wisconsin. The NRMSE of the proposed tensor quantization exponential regression algorithm was reduced by 0.0261, 0.0518, and 0.0387, for the test data in California, Texas, and Wisconsin. As shown in Table S4, the SMAPE of the proposed tensor quantization exponential regression algorithm was reduced by 0.0468, 0.0281, and 0.0075, for the test data from California, Texas, and Wisconsin.

We also aggregated the Walmart merchandising dataset by type of fresh produce and shop location. Then, we randomly selected ten fresh produce merchandising time series from the aggregated data. The ten fresh produce items are shown in Table S5, in which each fresh produce sales time series represents the same-day sales of one fresh produce in one shop. We used the first 1841 days and the last 100 days of fresh produce sales in the dataset as the input data and the validation data for the proposed method, respectively.

**Figure 4:**
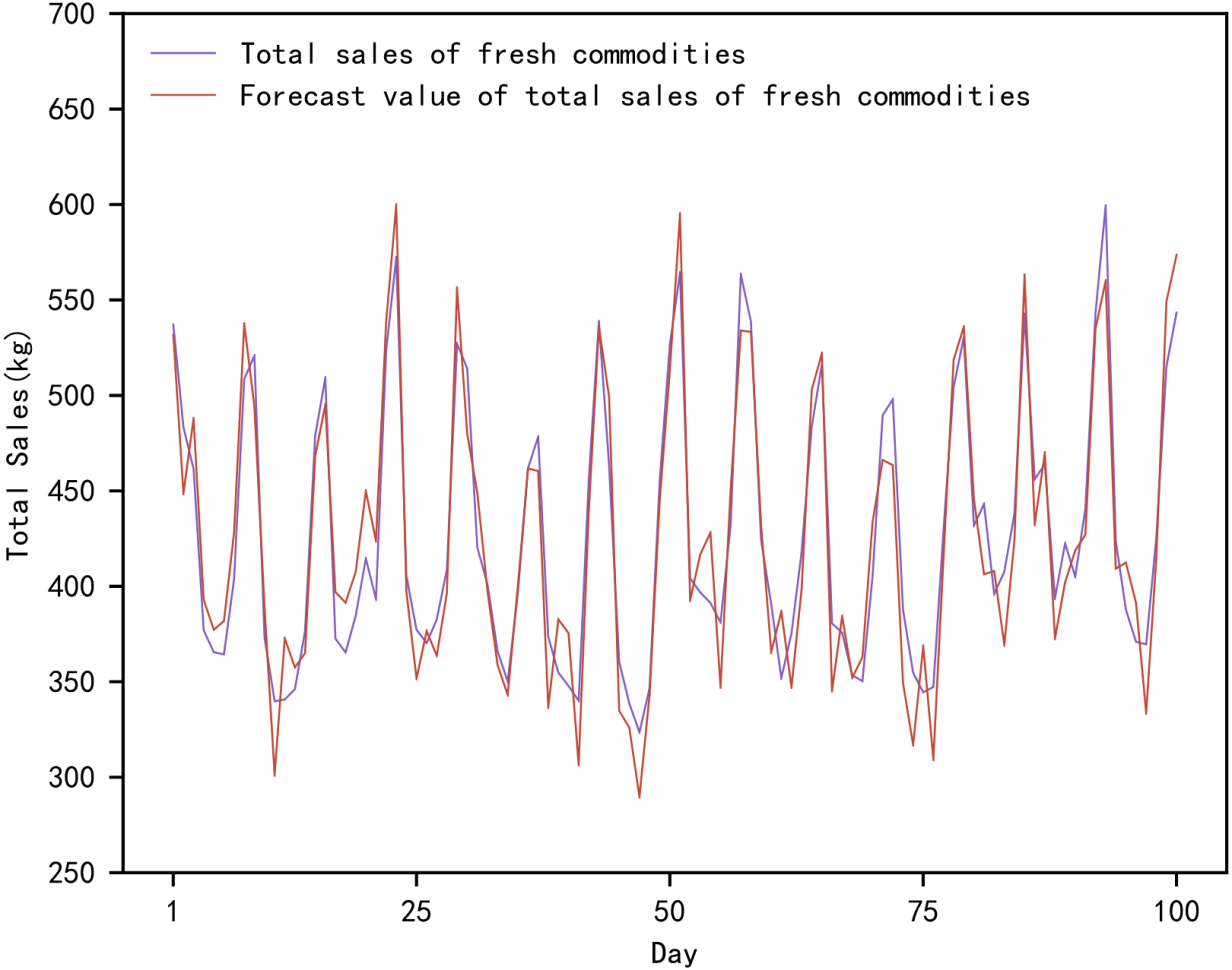
Comparison of true and predicted total sales of fresh commodities.

We used the proposed tensor quantization exponential regression algorithm and the cubic exponential regression algorithm for the last 100 days of fresh produce sales prediction, respectively. As shown in Table S5, the NRMSE of the proposed tensor quantization exponential regression algorithm is smaller than the classical cubic exponential regression algorithm for all ten randomly selected fresh produce sales.

Figure 4 shows the actual and predicted values of the total sales of fresh produce based on the proposed method. In Fig. 4, the actual total sales of fresh produce are the total value of the daily sales of the ten fresh produce mentioned above. The total sales forecast for fresh produce is the sum of the daily sales forecasts for the mentioned above ten fresh produce. The forecasts range from the day 1842 to the day 1941, in a total of 100 days.

### Model comparison

We also compare the values of NRMSE, SMAPE and R^2^ of the prediction results of ARIMA, VAR, MOAR, XGBoost, and deepAR for different proportions of the training set, as shown in Fig. S12.

As shown in Fig. S12, the horizontal axis represents the training set proportions. The vertical axes of (Figs. S12A–S12C) represent NRMSE, SMAPE, and R^2^ in sequence. For different training set proportions, NRMSE, SMAPE, and R^2^ of the proposed tensor quantization exponential regression algorithm outperformed the existing ARIMA, VAR, MOAR, XGBoost, and deepAR. Furthermore, in Fig. S12, NRMSE, SMAPE, and R^2^ all performed worse, when predicted by ARIMA, VAR, MOAR, XGBoost, and deepAR in the smaller proportions of the training set. In contrast, the proposed tensor quantization exponential regression algorithm had better performance for NRMSE, SMAPE, and R^2^ in the smaller proportions of the training set.

## Conclusions

A tensor quantization exponential regression algorithm was proposed for the supply forecasting and profiling of fresh goods in urban supermarket chains. In the proposed method, we first combined the tensor and cubic exponential regression algorithm models. Secondly, we tensorized the cubic exponential regression algorithm to predict multiple time series simultaneously and also improved the accuracy of the prediction. Finally, we used the Wal-Mart produce sales dataset as the experimental validation dataset. By comparing the proposed method with the existing ARIMA, VAR, MOAR, XGBoos, deepAR, and cubic exponential regression algorithms, the experimental results showed that the proposed method not only outperformed the above six existing methods but was also more stable.

In fact, the importance of supply forecasting is self-evident to individual consumers and caterers, as well as to other brick-and-mortar industries, services, and e-commerce, for social governance. Urban supermarket chains are key to reducing costs, improving efficiency and ensuring the quality and consistency of fresh commodities. The short shelf life of fresh commodities makes supply forecasting particularly important in the transport and distribution of fresh food. To achieve freshness, fresh commodities need to reach consumers quickly. The smaller the stock, the better supply forecasting, as fresh commodities are expensive to obtain in terms of freshness and safety. As a bridge between farmers and caterers, urban supermarket chains are centered on matching supply and demand. The ability to forecast the supply chain of fresh commodities determines the core competitiveness of urban supermarket chains in the future.

##  Supplemental Information

10.7717/peerj-cs.1138/supp-1 Supplemental Information 1 Transformation process of the multi-way delay embedding

10.7717/peerj-cs.1138/supp-2 Supplemental Information 2 Duplicate Matrix

10.7717/peerj-cs.1138/supp-3 Supplemental Information 3 Matrix expansion process: One-mode matrix expansion

10.7717/peerj-cs.1138/supp-4 Supplemental Information 4 Matrix expansion process: Two-mode matrix expansion

10.7717/peerj-cs.1138/supp-5 Supplemental Information 5 Matrix expansion process: Three-mode matrix expansion

10.7717/peerj-cs.1138/supp-6 Supplemental Information 6 High-order orthogonal iterations process

10.7717/peerj-cs.1138/supp-7 Supplemental Information 7 Tensor quantization third-order exponential regression algorithm

10.7717/peerj-cs.1138/supp-8 Supplemental Information 8 Inverse high-order orthogonal iterations process

10.7717/peerj-cs.1138/supp-9 Supplemental Information 9 Reverse multi-way delay embedding transform process

10.7717/peerj-cs.1138/supp-10 Supplemental Information 10 Duplication matrix

10.7717/peerj-cs.1138/supp-11 Supplemental Information 11 Urban supermarket chains commodities sales volume block Hankle tensor

10.7717/peerj-cs.1138/supp-12 Supplemental Information 12 Structure of urban supermarket chains commodities sales volume

10.7717/peerj-cs.1138/supp-13 Supplemental Information 13 Fresh commodities sales volume trend

10.7717/peerj-cs.1138/supp-14 Supplemental Information 14 Comparison of experimental results with different training set ratios Values of NRMSE of different train set ratios.

10.7717/peerj-cs.1138/supp-15 Supplemental Information 15 Comparison of experimental results with different training set ratios Values of SMAPE of different train set ratios.

10.7717/peerj-cs.1138/supp-16 Supplemental Information 16 Comparison of experimental results with different training set ratios Values of R^2^ of different train set ratios.

10.7717/peerj-cs.1138/supp-17 Supplemental Information 17 Partial data of x

10.7717/peerj-cs.1138/supp-18 Supplemental Information 18 High-order orthogonal iterations process

10.7717/peerj-cs.1138/supp-19 Supplemental Information 19 Comparison of NRMSE values

10.7717/peerj-cs.1138/supp-20 Supplemental Information 20 Comparison of SMAPE values

10.7717/peerj-cs.1138/supp-21 Supplemental Information 21 Comparison of NRMSE values of the two models

## Additional Information and Declarations

### Data Availability

The following information was supplied regarding data availability: The code and raw data are available at Figshare: li (2022): code. figshare. Software. https://doi.org/10.6084/m9.figshare.20831716.v1
li (2022): dataset. figshare. Dataset. https://doi.org/10.6084/m9.figshare.20831521.v1.
