# Supplementary figures and images for: Supply forecasting and profiling of urban supermarket chains based on tensor quantization exponential regression for social governance
Source: PeerJ Comput Sci. 2022 Nov 7;8:e1138. doi: 10.7717/peerj-cs.1138 (PMC9680888; doi:10.7717/peerj-cs.1138)

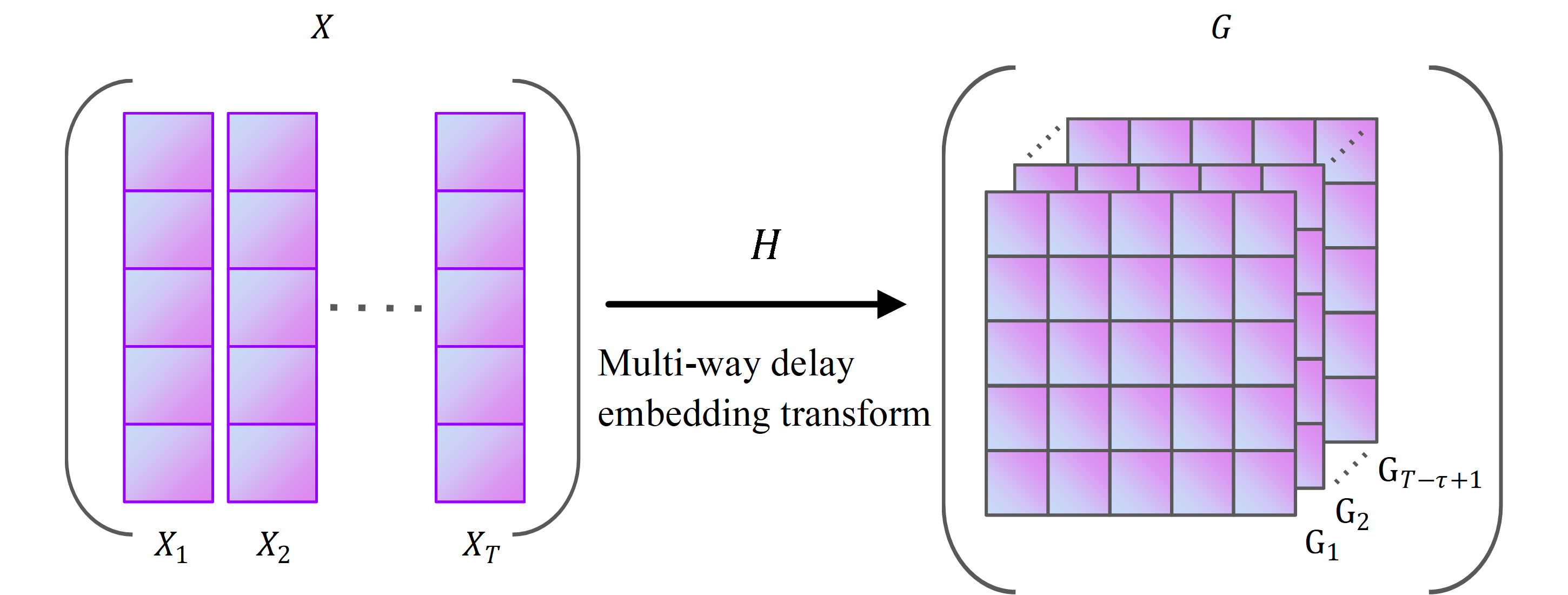

Supplement: Supplemental Information 1 [file peerj-cs-08-1138-s001.png]

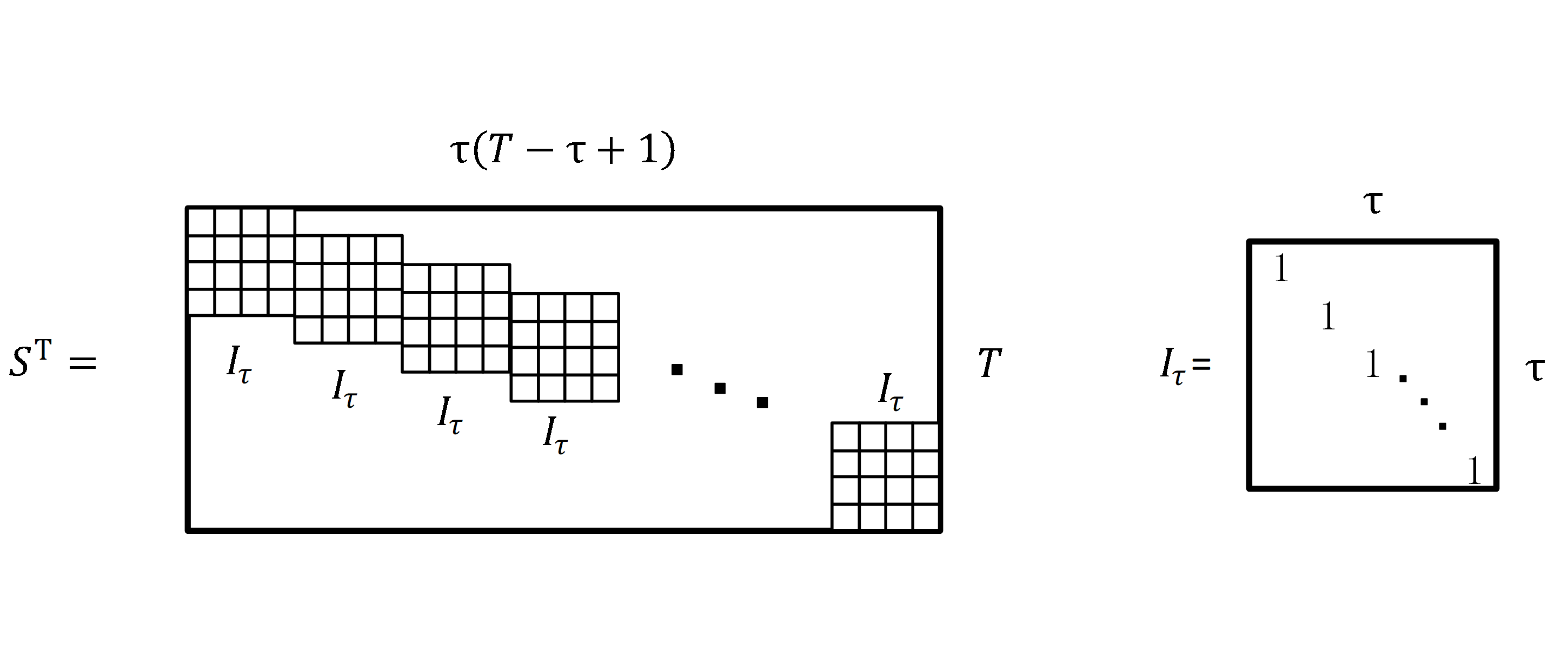

Supplement: Supplemental Information 2 [file peerj-cs-08-1138-s002.png]

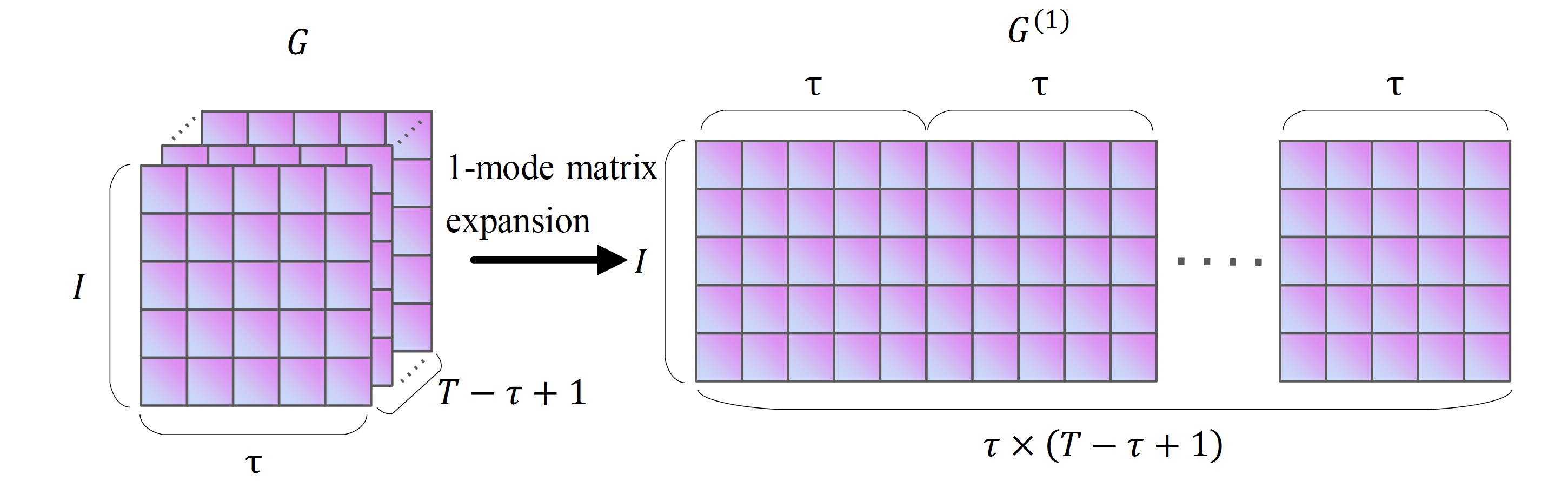

Supplement: Supplemental Information 3 [file peerj-cs-08-1138-s003.png]

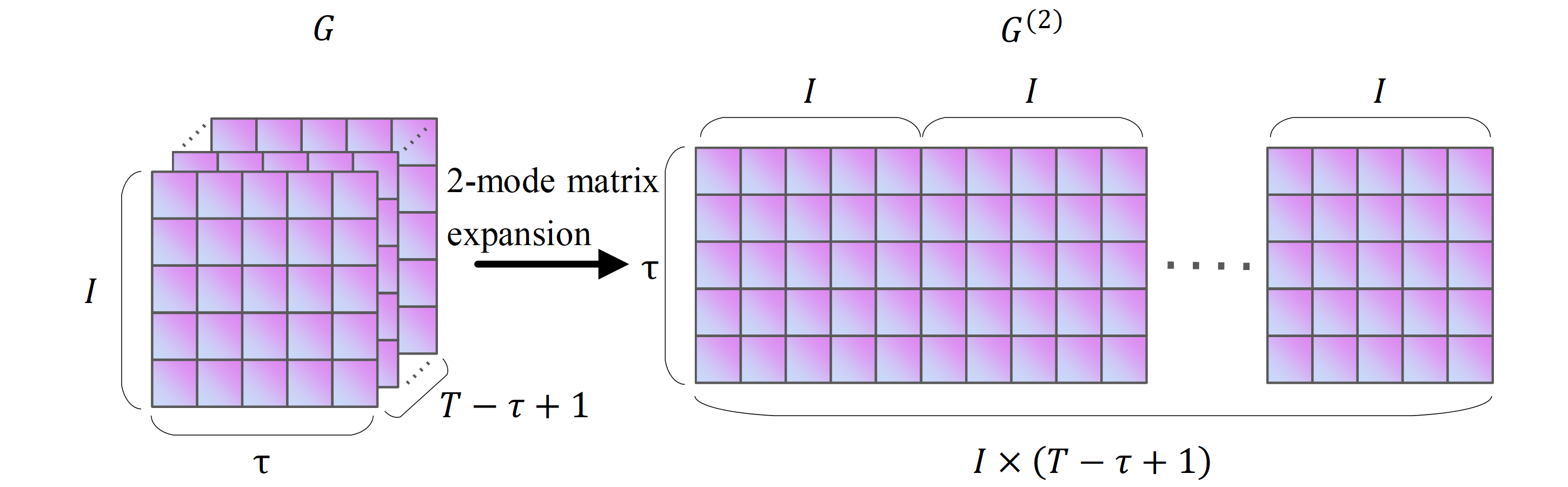

Supplement: Supplemental Information 4 [file peerj-cs-08-1138-s004.png]

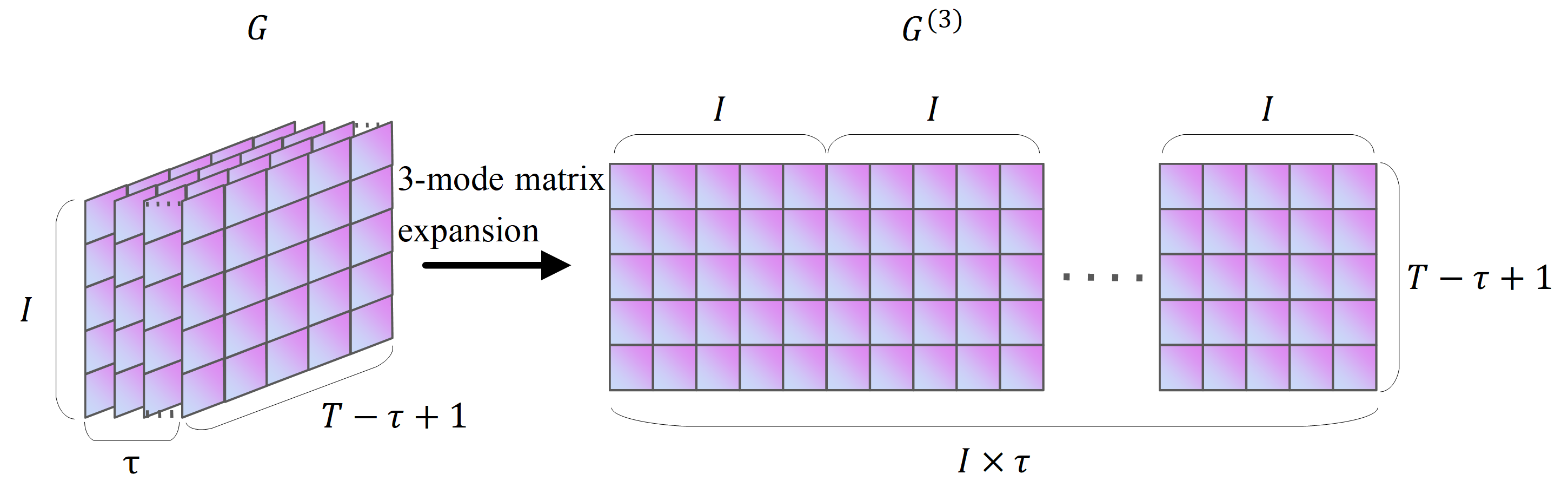

Supplement: Supplemental Information 5 [file peerj-cs-08-1138-s005.png]

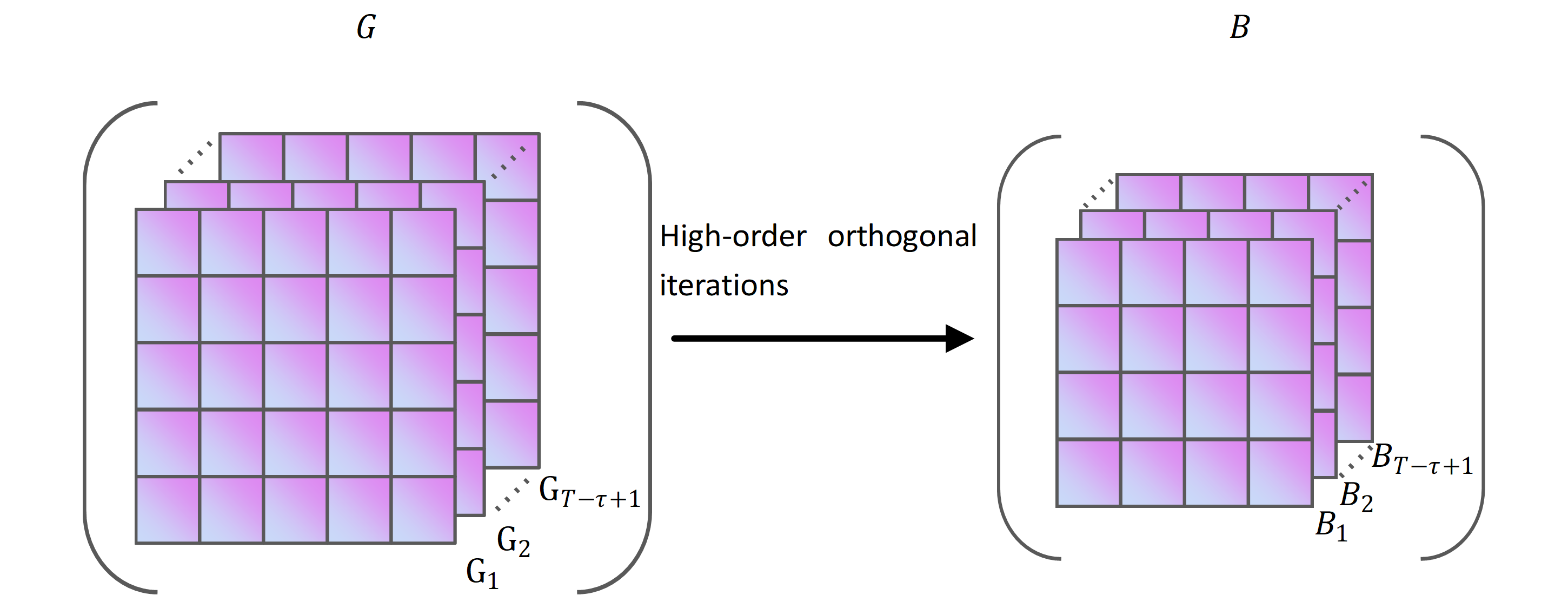

Supplement: Supplemental Information 6 [file peerj-cs-08-1138-s006.png]

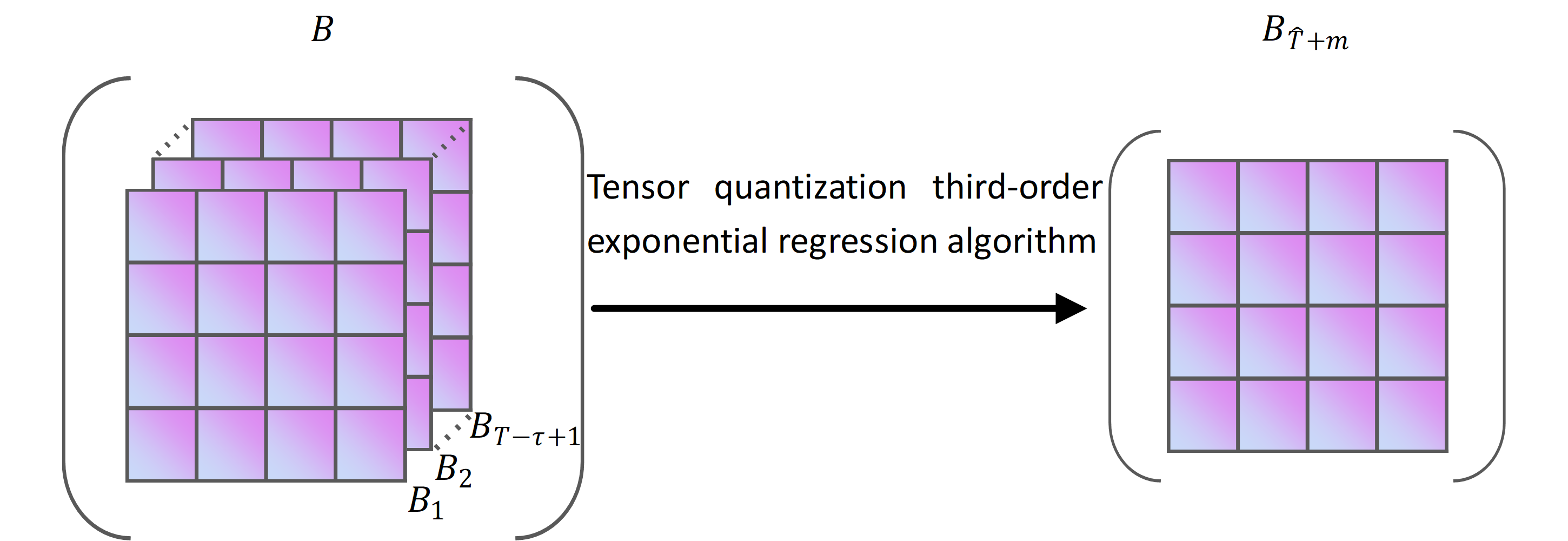

Supplement: Supplemental Information 7 [file peerj-cs-08-1138-s007.png]

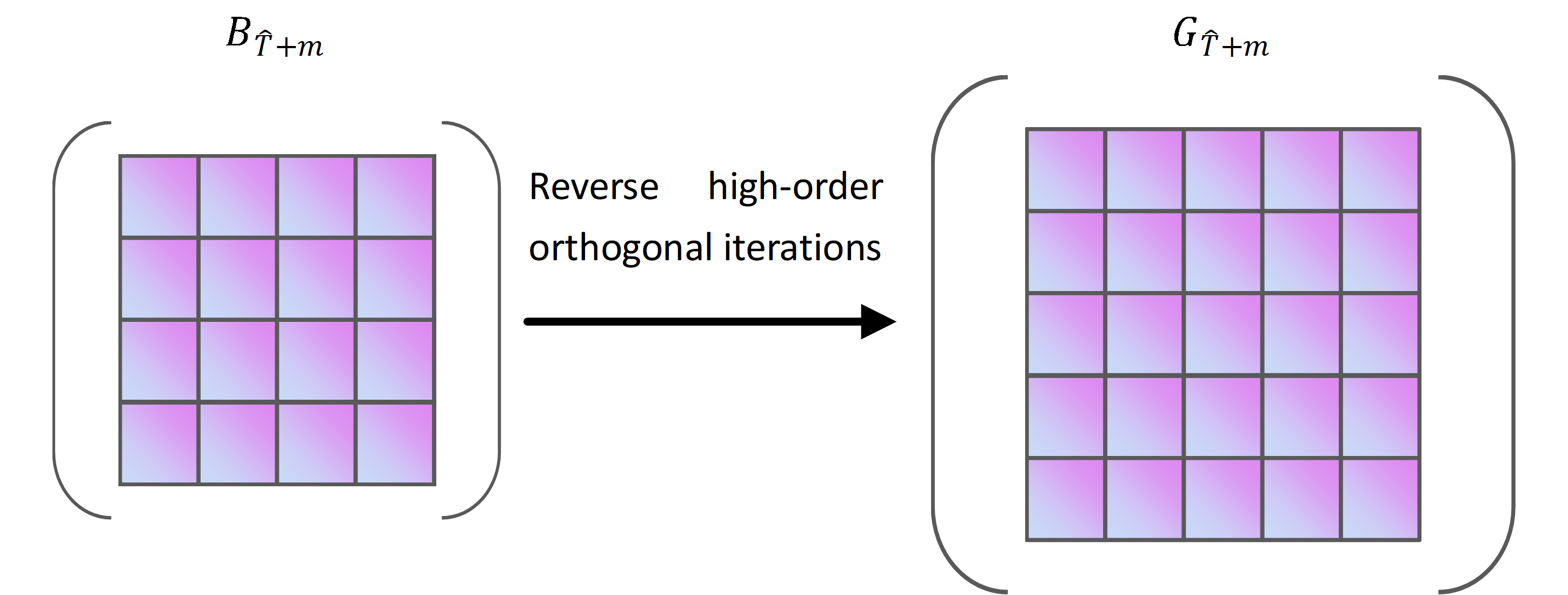

Supplement: Supplemental Information 8 [file peerj-cs-08-1138-s008.png]

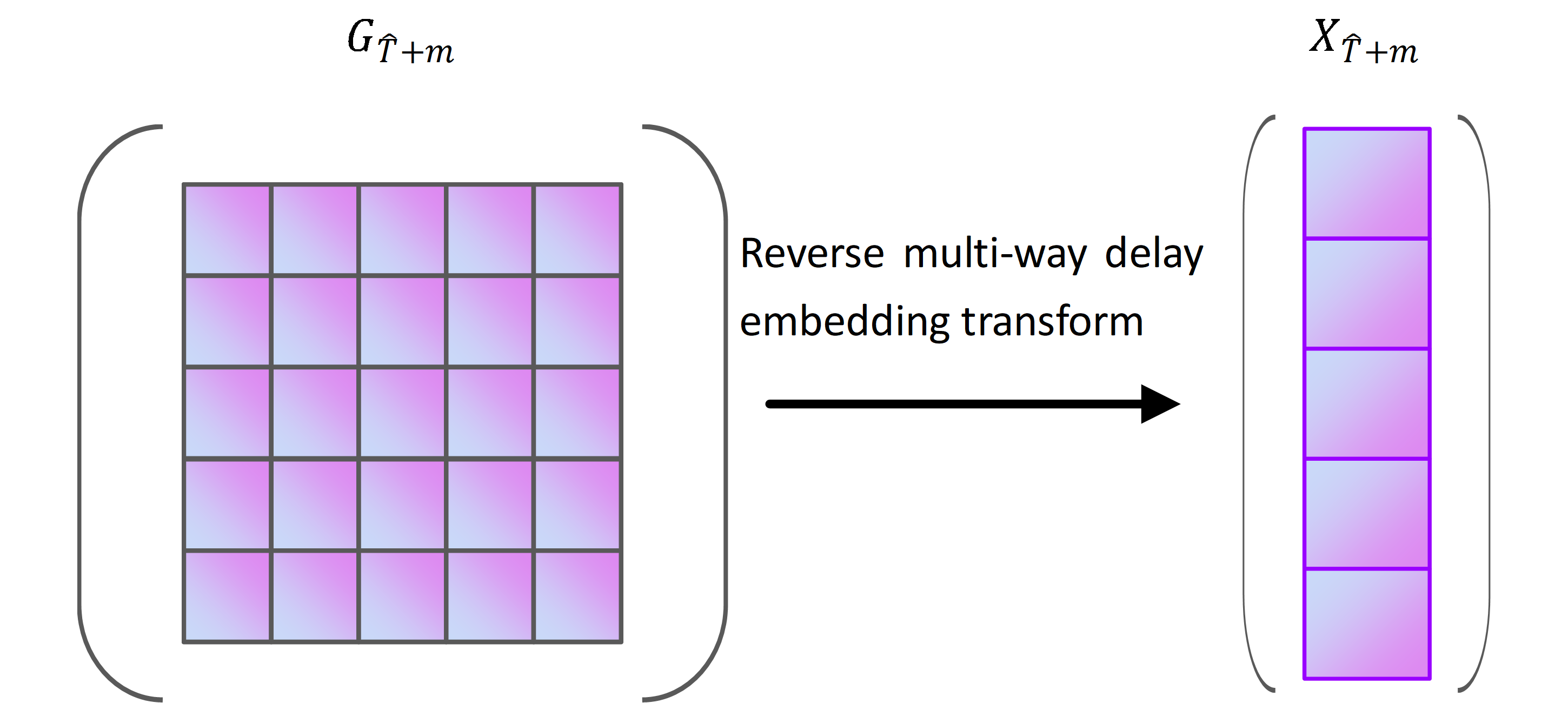

Supplement: Supplemental Information 9 [file peerj-cs-08-1138-s009.png]

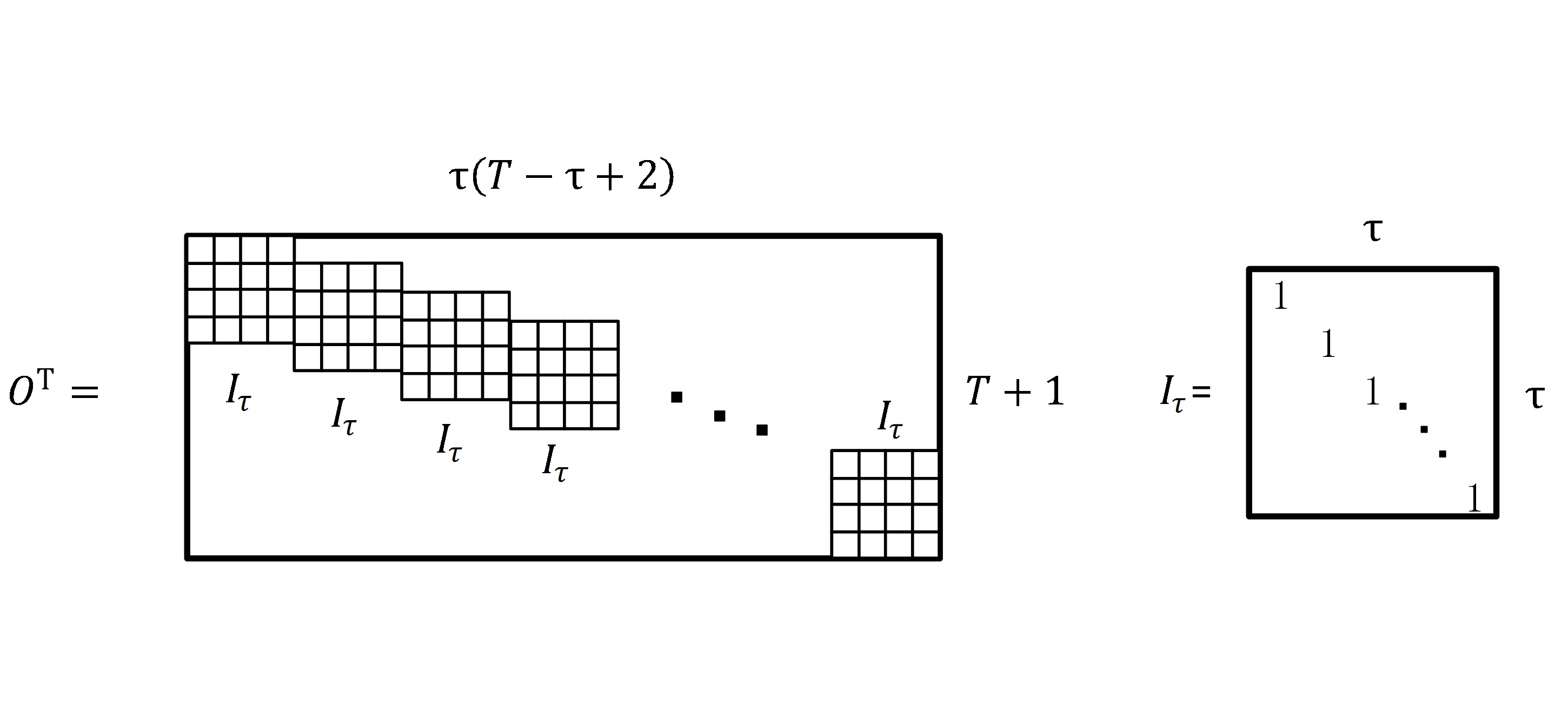

Supplement: Supplemental Information 10 [file peerj-cs-08-1138-s010.png]

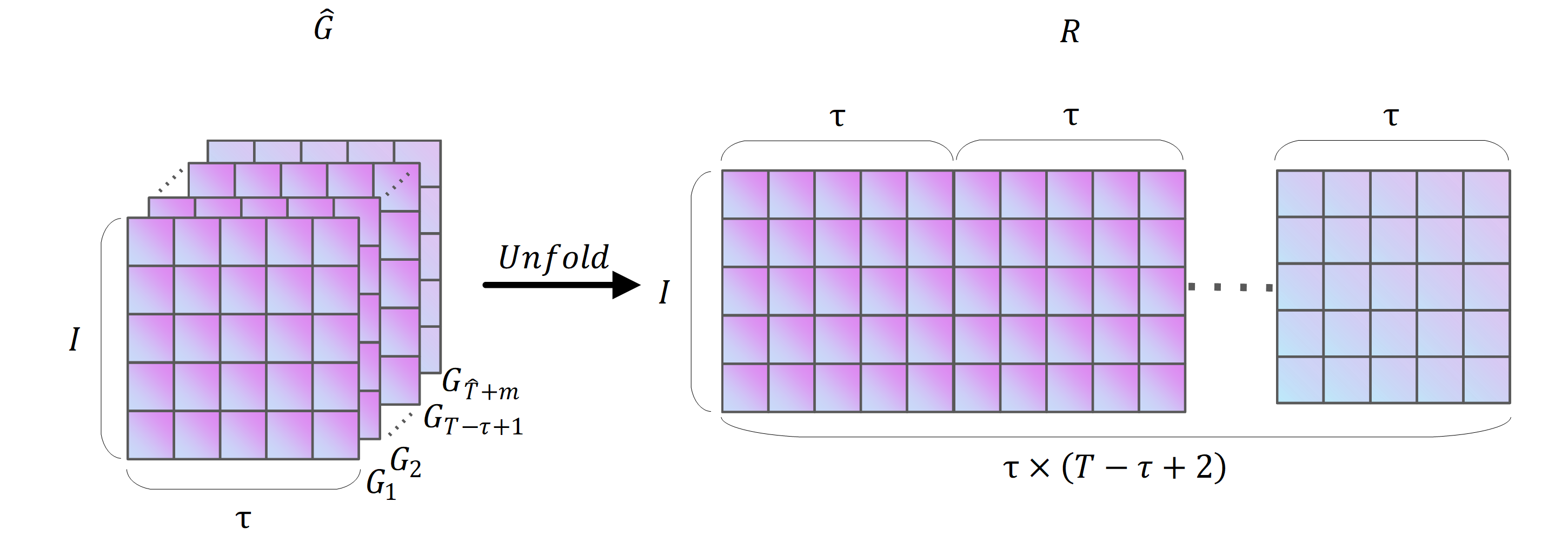

Supplement: Supplemental Information 11 [file peerj-cs-08-1138-s011.png]

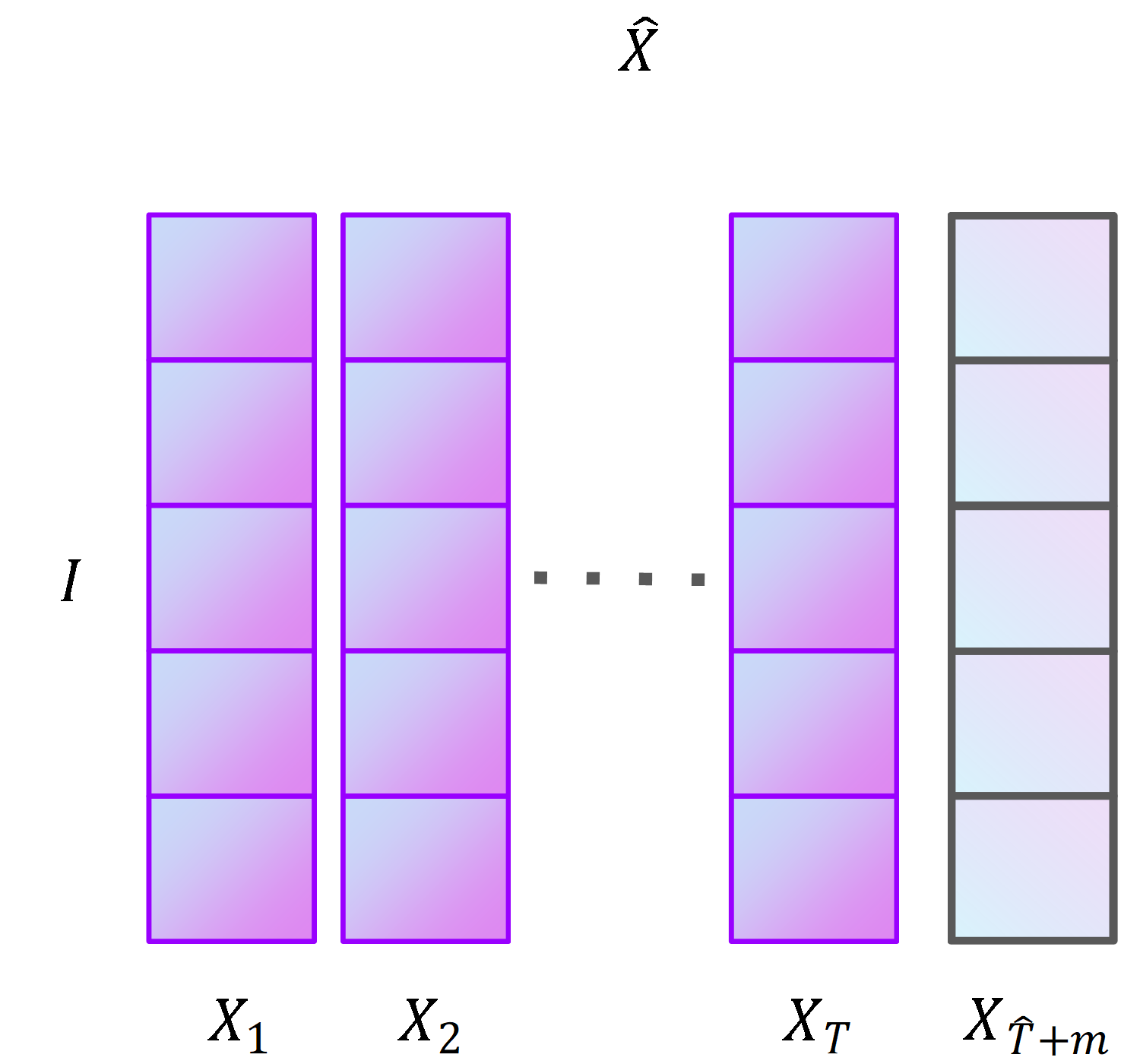

Supplement: Supplemental Information 12 [file peerj-cs-08-1138-s012.png]

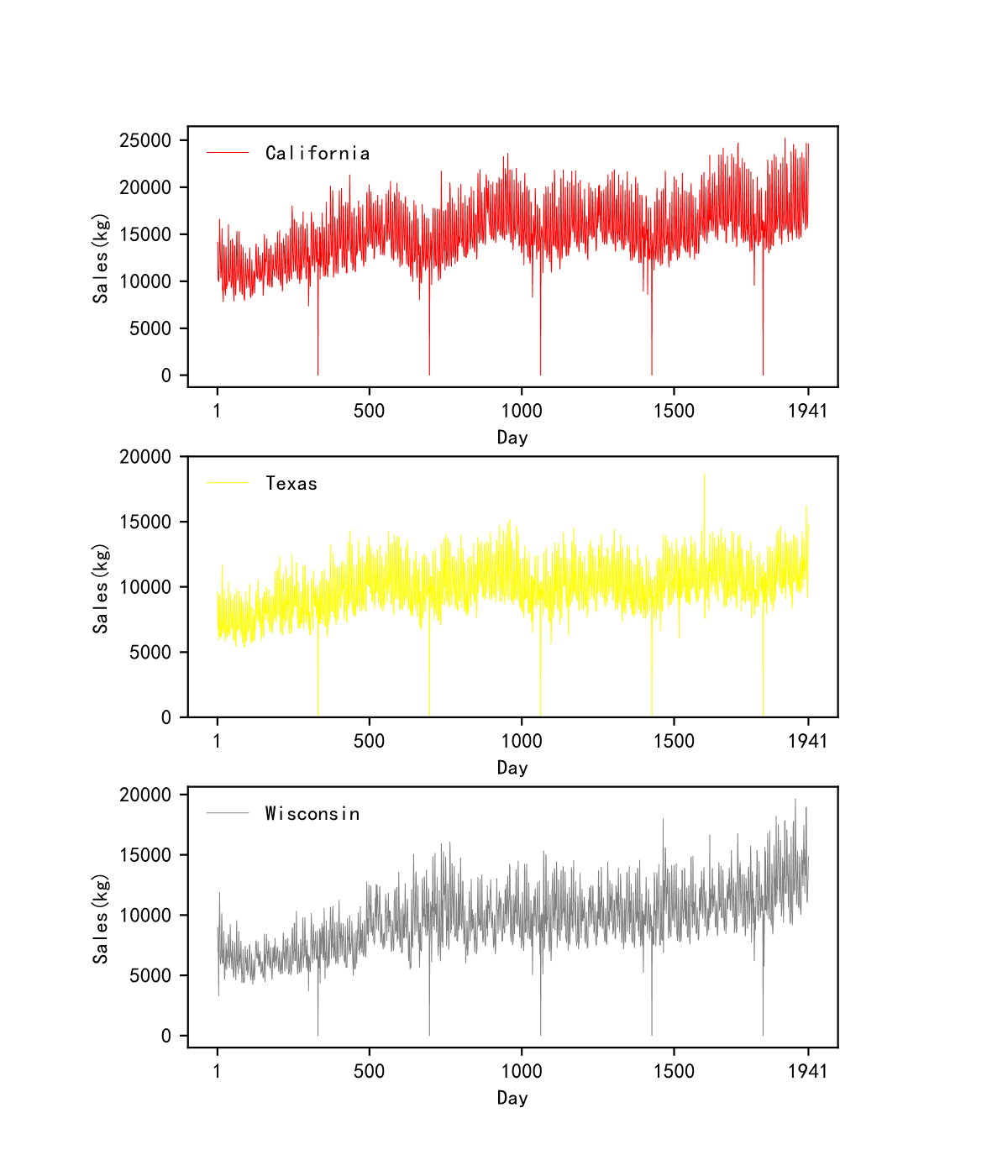

Supplement: Supplemental Information 13 [file peerj-cs-08-1138-s013.png]

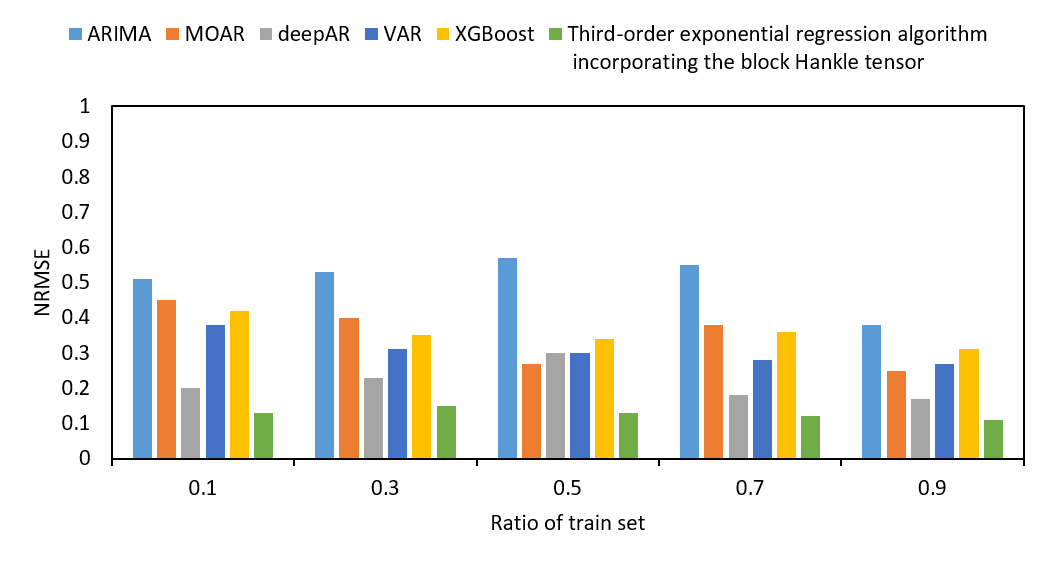

Supplement: Supplemental Information 14 — Values of NRMSE of different train set ratios. [file peerj-cs-08-1138-s014.png]

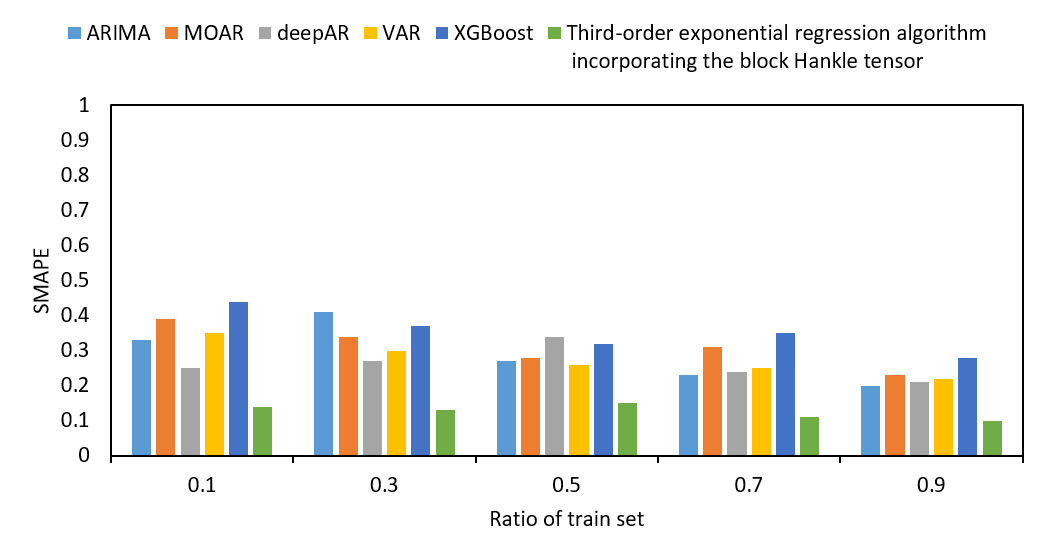

Supplement: Supplemental Information 15 — Values of SMAPE of different train set ratios. [file peerj-cs-08-1138-s015.png]

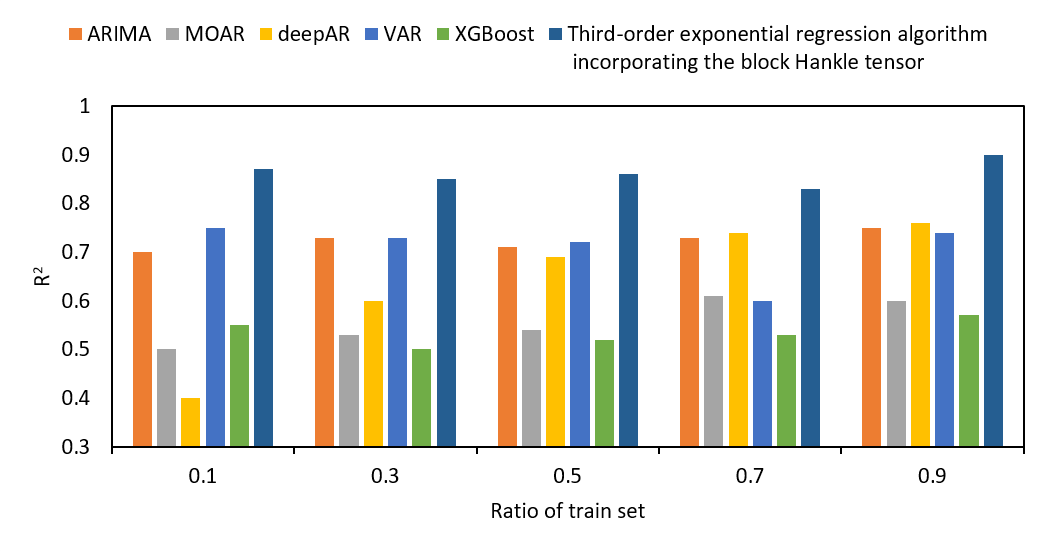

Supplement: Supplemental Information 16 — Values of R2 of different train set ratios. [file peerj-cs-08-1138-s016.png]
