# Supplementary material for: Supply forecasting and profiling of urban supermarket chains based on tensor quantization exponential regression for social governance
Source: PeerJ Comput Sci. 2022 Nov 7;8:e1138. doi: 10.7717/peerj-cs.1138 (PMC9680888; doi:10.7717/peerj-cs.1138)
Supplement: Supplemental Information 17 [file peerj-cs-08-1138-s017.docx]

Supplemental Table S1:

Partial data of x.

| state_id | day_1 | day_2 | day_3 | day_4 | day_5 |
| --- | --- | --- | --- | --- | --- |
| California | 14195 | 13805 | 10108 | 11047 | 9925 |
| Texas | 9438 | 9630 | 6778 | 7381 | 5912 |
| Wisconsin | 8998 | 8314 | 6897 | 6984 | 3309 |
