# Supplementary material for: Supply forecasting and profiling of urban supermarket chains based on tensor quantization exponential regression for social governance
Source: PeerJ Comput Sci. 2022 Nov 7;8:e1138. doi: 10.7717/peerj-cs.1138 (PMC9680888; doi:10.7717/peerj-cs.1138)
Supplement: Supplemental Information 18 [file peerj-cs-08-1138-s018.docx]

Supplemental Table S2:

High-order orthogonal iterations process.

| High-order orthogonal iterations |
| --- |
| 1. Truncate the high-order singular value decomposition to get the initial values of $U^{\left(k\right)}\left(k=1,2,3\right)$:  ,  , .  2. $K=1,2,\cdots,n$:  2.1 Computed the high-order singular value decomposition of the matrix $G^{\left(1\right)}\left(U_{k}^{\left(2\right)}\otimes U_{k}^{\left(3\right)}\right)$, and then selected the left singular vector corresponding to the $r_{1}$ largest singular values, and used the standard orthogonal basis of this space as the column vector of $U_{k+1}^{\left(1\right)}$.  2.2 Computed the high-order singular value decomposition of the matrix $G^{\left(2\right)}\left(U_{k}^{\left(3\right)}\otimes U_{k+1}^{\left(1\right)}\right)$, and then selected the left singular vector corresponding to the $r_{2}$ largest singular values, and used the standard orthogonal basis of this space as the column vector of $U_{k+1}^{\left(2\right)}$.  2.3 Computed the high-order singular value decomposition of the matrix $G^{\left(3\right)}\left(U_{k+1}^{\left(1\right)}\otimes U_{k+1}^{\left(2\right)}\right)$, and then selected the left singular vector corresponding to the $r_{3}$ largest singular values, and used the standard orthogonal basis of this space as the column vector of $U_{k+1}^{\left(3\right)}$.  3. Reach the maximum number of iterations $n$ cutoff.  4. $U^{\left(1\right)}=U_{k+1}^{\left(1\right)}$ ，$U^{\left(2\right)}=U_{k+1}^{\left(2\right)}$ ，$U^{\left(3\right)}=U_{k+1}^{\left(3\right)}$  5. Calculate the core matrix $B=G\times_{1}U^{\left(1\right)^{\text{T}}}\times_{2}U^{\left(2\right)^{\text{T}}}\times_{3}U^{\left(3\right)^{\text{T}}}$. |
