# Supplementary material for: Supply forecasting and profiling of urban supermarket chains based on tensor quantization exponential regression for social governance
Source: PeerJ Comput Sci. 2022 Nov 7;8:e1138. doi: 10.7717/peerj-cs.1138 (PMC9680888; doi:10.7717/peerj-cs.1138)
Supplement: Supplemental Information 20 [file peerj-cs-08-1138-s020.docx]

Supplemental Table S4:

Comparison of SMAPE values.

| Time Series Name | California | Texas | Wisconsin |
| --- | --- | --- | --- |
| third-order exponential regression algorithm | 0.1373 | 0.1106 | 0.1354 |
| third-order exponential regression algorithm incorporating the block Hankle tensor | 0.0905 | 0.0825 | 0.1279 |
