# Supplementary material for: Supply forecasting and profiling of urban supermarket chains based on tensor quantization exponential regression for social governance
Source: PeerJ Comput Sci. 2022 Nov 7;8:e1138. doi: 10.7717/peerj-cs.1138 (PMC9680888; doi:10.7717/peerj-cs.1138)
Supplement: Supplemental Information 21 [file peerj-cs-08-1138-s021.docx]

Supplemental Table S5:

Comparison of NRMSE values of the two models.

| Fresh commodities name | Third-order exponential regression algorithm | Third-order exponential regression algorithm incorporating the block Hankle tensor |
| --- | --- | --- |
| Apple | 0.1432 | 0.1296 |
| Pear | 0.1374 | 0.1134 |
| Egg | 0.1352 | 0.1247 |
| Chicken | 0.1518 | 0.1107 |
| Beef | 0.1349 | 0.1024 |
| Fish | 0.1296 | 0.0930 |
| Shrimp | 0.1708 | 0.1294 |
| Carrot | 0.1425 | 0.1202 |
| Green vegetables | 0.1386 | 0.1136 |
| Western Bluebell | 0.1502 | 0.1093 |
